# Neuropathological signatures revealed by transcriptomic and proteomic analysis in *Pten*-deficient mouse models

**DOI:** 10.1038/s41598-023-33869-7

**Published:** 2023-04-25

**Authors:** Stanley K. K. Cheung, Jacinda Kwok, Penelope M. Y. Or, Chi Wai Wong, Bo Feng, Kwong Wai Choy, Raymond C. C. Chang, J. Peter H. Burbach, Alfred S. L. Cheng, Andrew M. Chan

**Affiliations:** 1 School of Biomedical Sciences, The Chinese University of Hong Kong, Hong Kong, SAR China; 2 Department of Obstetrics and Gynaecology, The Chinese University of Hong Kong, Hong Kong, SAR China; 3 Laboratory of Neurodegenerative Diseases, School of Biomedical Sciences, Li Ka Shing Faculty of Medicine, The University of Hong Kong, Pokfulam, Hong Kong, SAR China; 4 Department of Translational Neuroscience, University Medical Center Utrecht, Utrecht, The Netherlands; 5 Brain and Mind Institute, The Chinese University of Hong Kong, 4/F, Hui Yeung Shing Building, Hong Kong, SAR China; 6 Present Address: Department of Pharmaceutical Sciences, University of Toronto, Toronto, Canada; 7 Present Address: Louvain Institute of Biomolecular Science and Technology, Université catholique de Louvain, Louvain-la-Neuve, Belgium

**Keywords:** Cell biology, Computational biology and bioinformatics, Developmental biology, Molecular biology, Neuroscience, Molecular medicine, Pathogenesis, Neurology, Neurodevelopmental disorders, Genetics, Functional genomics, Gene expression, Medical genetics, Neurodevelopmental disorders

## Abstract

PTEN hamartoma tumour syndrome is characterised by mutations in the human *PTEN* gene. We performed transcriptomic and proteomic analyses of neural tissues and primary cultures from heterozygous and homozygous *Pten*-knockout mice. The somatosensory cortex of heterozygous *Pten-*knockout mice was enriched in immune response and oligodendrocyte development Gene Ontology (GO) terms. Parallel proteomic analysis revealed differentially expressed proteins (DEPs) related to dendritic spine development, keratinisation and hamartoma signatures. However, primary astrocytes (ASTs) from heterozygous *Pten-*knockout mice were enriched in the extracellular matrix GO term, while primary cortical neurons (PCNs) were enriched in immediate-early genes. In ASTs from homozygous *Pten*-knockout mice, cilium-related activity was enriched, while PCNs exhibited downregulation of forebrain neuron generation and differentiation, implying an altered excitatory/inhibitory balance. By integrating DEPs with pre-filtered differentially expressed genes, we identified the enrichment of traits of intelligence, cognitive function and schizophrenia, while DEPs in ASTs were significantly associated with intelligence and depression.

## Introduction

PTEN hamartoma tumour syndrome (PHTS) is a spectrum of disorders caused by heterozygous germline mutations of the phosphatase and tensin homologue deleted on chromosome 10 (*PTEN*) tumour suppressor gene, which is a gatekeeper of the phosphoinositide 3-kinase (PI3K) pathway^1^. PHTS is characterised by a hamartomatous growth that develops in various parts of the body^2,3^ and neurodevelopmental disorders, such as macrocephaly, learning disabilities, developmental delays, and autism spectrum disorder (ASD)^4^. The prevalence of ASD in PHTS is estimated to be ~ 22%^5,6^ and the frequency of PTEN mutation in ASD patients with macrocephaly is ~ 10%^2,7^. The results of a recent clinical study suggest the disruption of frontal lobe systems, including full-scale IQ, attention, motor coordination, cognitive function and sensory deficits, in patients with PHTS^6^. Indeed, heterozygous *Pten-*knockout (*Pten*^+/−^) mice exhibit broad brain overgrowth with excess neurons at birth and cortical glia in adulthood, deficits in social behaviour, acquired repetitive behaviours^8–10^ and enhanced axonal branching and connectivity in projections of the medial prefrontal cortex to the basolateral amygdala axons^11^. Moreover, *CaMK2-Cre*^+/−^; *Pten*^+/loxP^ mice display reduced intrinsic excitability of pyramidal neurons due to increased expression levels of small-conductance calcium-activated potassium channels^12^. At the cellular level, the expression profile of neural precursor cells prepared from the subventricular zone (SVZ) of adult *Pten*^+/−^ mice shows increases in vascular endothelial growth factor and doublecortin (DCX) expression levels and a shift in DCX-positive cells from the SVZ to the olfactory bulb^13^.

Most of the other functions of PTEN in the brain have been discovered using conditional *Pten*-knockout mice due to embryonic lethality associated with homozygous germline *Pten* deletion^14^. One of the earliest mouse models using *GFAP-Cre*-mediated deletion of *Pten* in astrocytes (ASTs) and neurons in the whole brain resulted in macrocephaly, increased dendritic spine density, abnormal enlargement of synaptic structures, layering defects, seizures and ataxia^15–19^. To examine neuronal-specific effects, *Pten* was knocked out in dopaminergic neurons using dopamine transporter (*DAT*)*-Cre*^20,21^, in excitatory neurons in the forebrain using calcium/calmodulin-dependent protein kinase II alpha (*CamKIIα*)*-Cre*^22^, in Purkinje neurons using Purkinje cell protein 2 (*L7*)*-Cre*^23^ and in subsets of postmitotic neurons using neuron-specific enolase (*NSE*)*-Cre*^24–26^. These knockout strategies resulted in hypertrophic neurons with aberrant dendritic arborisation and increased axonal density and length. Electrophysiologically, *Pten* knockout driven by *CamKIIα-Cre* and *NSE-Cre* induces impairment of hippocampal long-term potentiation and metabotropic glutamate receptor-dependent long-term depression, respectively. Hence, specific behavioural phenotypes, such as impaired spatial memory, abnormal social interaction and abnormal response to sensory stimuli, have been assigned to specific subsets of neurons. In addition, mice with postnatally generated *Pten* knockout in hippocampal dentate granule cells exhibit hyperexcitability due to an increased excitatory synaptic drive^27–30^. Interestingly, *Pten* deletion in cortical γ-aminobutyric acid (GABA)ergic interneurons using NK2 homeobox 1 (*Nkx2.1*)*-Cre*, which targets medial and caudal ganglionic eminences and preoptic area progenitors, resulted in the preferential loss of somatostatin (SST)-positive interneurons, which increased the ratio of parvalbumin/SST interneurons and induced inhibitory actions on glutamatergic cortical neurons, leading to deficits in social behaviour^31^. The above evidence supports the hypothesis of disrupted excitatory/inhibitory (E/I) balance as a potential mechanism of autism^32^.

The loss of PTEN leads to the upregulation of mechanistic target of rapamycin (mTOR)-driven transcriptional and translational machinery^33,34^. Increased PI3-K/mTOR signalling has been implicated in the autistic phenotypes of PHTS patients, as treatment with the pharmacological mTOR inhibitor rapamycin normalises some of the behavioural defects in *Pten*-knockout mice^35^. However, the PTEN-regulated genes responsible for the neuropathological or ASD phenotypes in PHTS patients remain largely unknown. Furthermore, homozygous *PTEN* mutations have not been found in PHTS patients and findings in conditional *Pten*-knockout models have not been reproduced in heterozygous *Pten-*knockout models, which imply that the heterozygous loss of PTEN may have very subtle pathological consequences that could not be detected using conventional methods.

In this report, we present a comprehensive analysis of the proteomes and transcriptomes of the somatosensory cortex, which is a region responsible for receiving and processing sensory stimuli, and has been implicated in ASD^36^. This region develops actively during the early postnatal period, as determined by previous studies of the E/I balance regulatory networks^37,38^ in heterozygous *Pten-*knockout mice. Moreover, we dissected the cell-type-specific contribution from the transcriptomes of primary neural progenitor cells (NPCs), cortical ASTs, and primary cortical neurons (PCNs) derived from heterozygous and homozygous *Pten*-knockout mice. Our findings may provide the underlying molecular mechanism responsible for ASD phenotypes observed in PHTS patients.

## Methods

### Mice

The permission to conduct animal experimentation was obtained from The Department of Health of the Government of the Hong Kong Special Administrative Region, under the following two animal licences (20-71) in DH/HT&A/8/2/1 Pt.4 and (20-72) in DH/HT&A/8/2/1 Pt.4. All animal protocols were approved by the Animal Experimentation and Ethics Committee of the Chinese University of Hong Kong under the approval number, 14/148/MIS. All methods are reported in accordance with ARRIVE guidelines and were carried out in accordance with relevant guidelines and regulations. All the mice were maintained under selective pathogen-free conditions. All mice had a C57BL/6J background (see Supplementary Table ST4 for a summary). B6.129-Pten^tm1Rps^/Nci heterozygous (*Pten*^+/−^) mice were obtained from the National Cancer Institute’s Mouse Models of Human Cancers Consortium (Frederick, MD, USA)^14^. The *Pten*^+/−^ mice were crossed with wild-type (WT) C57BL/6J mice to generate either WT or *Pten*^+/−^ progeny. B6.129S4-*Pten*^tm1Hwu^/J (*Pten*^fl/fl^) and B6.Cg-Tg(*Nes-Cre*)1Kln/J (*Nes-Cre*) mice were purchased from the Jackson Laboratory (Bar Harbor, ME, USA)^39,40^. *Pten*^fl/fl^ mice were crossed with *Nestin* promoter-driven *Cre* recombinase (*Nes-Cre*) mice to generate compound heterozygous mice (*Nes-Cre*:*Pten*^fl/+^). *Nes-Cre*:*Pten*^fl/+^ mice were then mated with *Pten*^fl/fl^ mice to generate the following four genotypes: *Pten*^fl/+^ (*Ctrl*), *Pten*^fl/fl^ (*Ctrl*), *Nes-Cre*:*Pten*^fl/+^ (*Nes*-*HET*) and *Nes-Cre*:*Pten*^fl/fl^ (*Nes*-*KO*). All mice were euthanized with CO_2_, except for P0 and P2 pups which were euthanized with isoflurane.

### Primary cultures

Primary NPCs, PCAs and PCNs were established based on previously published protocols^41^. The detailed procedures are provided in the Supplementary Information.

### Brain microdissection

Mice were perfused with 30 mL of ice-cold phosphate-buffered saline. The SSC (1.82 mm to 1.94 mm anteroposterior from the bregma) was dissected under a stereomicroscope (SMZ660; Nikon, Tokyo, Japan)^42^. The right hemisphere SSC was snap-frozen in liquid nitrogen, while the left hemisphere SSC was triturated with 1 mL of TRIzol™ Reagent (Thermo Fisher Scientific, Waltham, MA) before snap freezing.

### RNA-seq

Total RNA was extracted using TRIzol™ Reagent. Three mice per group were used as biological replicates, except for P30M mice, of which there were only two in the *Pten*^+/−^ group. The RNA samples were subjected to quantitative RNA-seq by BGI (Hong Kong SAR) or GROKEN Bioscience (Hong Kong SAR). Briefly, polyA-enriched mRNA was reverse-transcribed into cDNA and purified using magnetic beads. Adaptors were ligated to the DNA ends, size fractionated and enriched by polymerase chain reaction (PCR) amplification. The quality of the DNA fragments was determined using an Agilent 2100 Bioanalyzer (Agilent, Santa Clara, CA, USA) and sequenced on a HiSeq2500, HiSeq4000 or X Ten platform (Illumina, San Diego, CA, USA). Further information on data analysis is provided in the Supplementary Information section. Raw data and normalised counts are available on the Gene Expression Omnibus database under series GSE190879.

### Proteomic analysis

Snap-frozen SSCs were processed for proteomics analysis based on a previously published protocol^43^. Approximately 100 µg of soluble protein was subjected to iTRAQ analysis (BGI). Additional experimental procedures and raw tandem mass spectrometry data are provided in the Supplementary Information section. The mass spectrometry proteomics data have been deposited to the ProteomeXchange Consortium via PRIDE, under the identifier PXD030573^44^.

### Quantitative reverse transcription PCR

First-strand cDNA was generated from 0.5 μg of DNase I (Thermo Fisher)-treated total RNA using the High-Capacity cDNA Reverse Transcription Kit (Thermo Fisher Scientific) based on the manufacturer’s instructions. Quantitative PCR was performed using the Power SYBR Green PCR Master Mix (Thermo Fisher Scientific) and the ABI QuantStudio 7 Flex Real-Time PCR System (384-well plates, Thermo Fisher Scientific). Primers were designed using the online tool GETprimer (https://gecftools.epfl.ch/getprime). Only intron-spanning primers that also targeted protein-coding transcripts were selected. The specific primers used in this analysis are listed in the supplementary information section. Cycle threshold (CT) values were first normalised to the expression of β-actin, and relative gene expression levels were calculated using the 2^−∆∆CT^ method.

### Integration with ASD co-expression modules

Gene lists of ASD co-expression modules were obtained from Parikshak et al*.*^45^. The enrichment of the identified DEGs in the SSC within the ASD co-expression modules was calculated using a cumulative hypergeometric test, and the FDR was adjusted using the Benjamini–Hochberg correction for multiple comparisons.

### Integration with neurological disease GWAS data and neurobehavioural traits

GWAS catalogue version 1.0.2 was used for analysis (https://www.ebi.ac.uk/gwas/home). Risk genes for the following neuropathologies and neurobehavioural traits were extracted from the raw file: Alzheimer’s disease, Parkinson’s disease, amyotrophic lateral sclerosis, multiple sclerosis, autism, schizophrenia, bipolar disorder, attention deficit hyperactivity disorder, depression, anxiety, language disorder, intelligence and cognitive function. Duplicated genes, non-protein coding genes, GWAS loci in intergenic regions and genes with a *p*-value > 9 × 10^–6^ were removed. Genes reported in ‘MAPPED_GENE’ were used only if the ‘REPORTED GENE(S)’ field was empty. Statistical significance between the GWAS gene lists and our DEP and DEG lists against the total number of protein-coding genes (GENCODE Release version 38) as background was calculated using a cumulative hypergeometric test, and the FDR was adjusted using Benjamini–Hochberg correction for multiple comparisons.

### Statistical analysis

For western blotting and reverse transcription-quantitative PCR analyses, statistically significant differences between two groups were identified using an unpaired Student’s t-test. Comparisons of age, sex and genotype effects in the SSC were made using a three-way analysis of variance, followed by a Bonferroni post hoc multiple comparisons test. Statistical significance was defined as a *p*-value < 0.05. The fold change (FC) directionality of DEGs and DEPs in the SSC and the log_2_FC of DEGs in primary cultures of PCNs and ASTs from *Pten*^+/−^ and *Nes-KO* mice (FDR < 0.05) were calculated using Spearman’s correlation test.

### Ethics approval and consent to participate

All of the animal procedures were approved by the Animal Experimentation and Ethics Committee of the Chinese University of Hong Kong and maintained under selective pathogen-free conditions.

## Results

### Transcriptomic analysis reveals increased immune response and oligodendrocyte development in somatosensory cortex

To investigate the effect of the heterozygous loss of *Pten* on the somatosensory cortex (SSC) at different ages, the activation state of the PI3K/mTOR signalling pathway was first characterised. Postnatal day 30 and 42 (P30 and P42) were selected for analysis, as higher spine density was observed in P29-30 *Tsc2*-null mice^46^, and autistic phenotypes were reported in multiple PTEN knockout mouse models from 6 week (P42) onward^9,47^. However, this analysis failed to reveal significant changes except for the elevated levels of p-S6. (Supplementary Fig. 1A,B).

**Figure 1:**
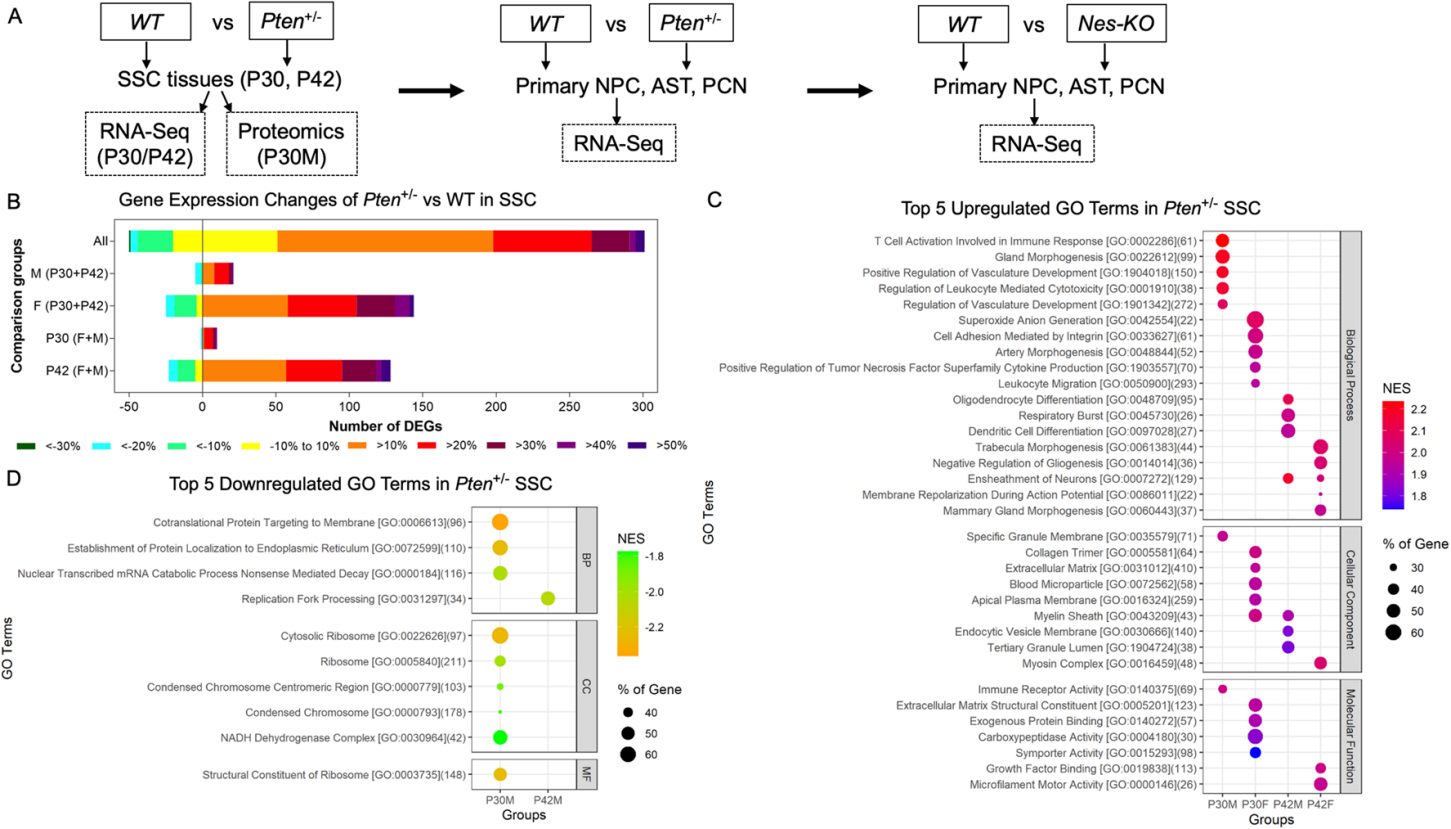
Transcriptomic analysis reveals increased immune response and oligodendrocyte development in somatosensory cortex. (**A**) Workflow of current study summarizing the genotypes, stages, cell types, and molecular techniques used. (**B**) Number of protein coding genes that were differentially expressed (false discovery rate < 0.05) in the somatosensory cortex of male (M) and female (F) *Pten*^+/−^ mice at P30 ( male: n=3 for wild-type, and n=2 for *Pten*^+/−^; female: n=3 for wild-type and *Pten*^+/−^) and P42 (n = 3 for each group). Differentially expressed genes (DEGs) are stratified by their percentage fold change (*Pten*^+/−^ vs. wild-type). Age and sex-independent analysis after pooling P30M, P30F, P42M and P42F groups; M (P30 + P42), male-specific analysis after pooling P30M and P42M groups; F (P30 + P42), female-specific analysis; P30 (F + M), P30-specific analysis after pooling P30F and P30M groups; P42 (F + M), P42-specific analysis after pooling P42F and P42M groups. (**C**) Top five upregulated Gene Ontology (GO) terms in each group. (**D**) Top five downregulated GO terms in each group.

It is possible that global transcriptomic and proteomic changes could be responsible for the ASD phenotypes in PHTS. We established a workflow to delineate these molecular changes in *Pten*-deficient mice (Fig. 1A). To delineate the molecular changes in the SSC of *Pten*^+/−^ mice, RNA-sequencing (RNA-seq) data were analysed using the DESeq2 software package. Irrespective of age and sex, there were 351 differentially expressed genes (DEGs), of which 301 were upregulated and 50 were downregulated in *Pten*^+/−^ mice compared with their littermate controls (Fig. 1B). Of the 10 most significant DEGs, eight were upregulated (Fig. 1B). *ADGRG1*, *LGI3* and *NEFM* are known to play roles in neural development and are associated with neurological diseases, such as epilepsy (Supplementary Fig. 1C). Additional upregulated genes, such as *C1QC* and *CSF1R*, are known immune-related genes^48,49^. *RPRM*, which is involved in the p53-dependent G2 arrest of the cell cycle, was downregulated^50^. Furthermore, relatively more DEGs were observed in the following two groups—female mice, irrespectively of age, and mice at P42, irrespective of sex (Fig. 1B; Supplementary Fig. 1D–H).

Gene Ontology (GO) and canonical pathway enrichment analyses revealed that the immune response and vasculature development were amongst the top upregulated pathways in P30 male (P30M) mice (Fig. 1C; Supplementary Fig. 1I,J). Similarly enriched terms, such as artery morphogenesis, cytokine production and leukocyte migration, were upregulated in P30 female (P30F) mice (Fig. 1C). Immune-related terms, such as dendritic cell differentiation, were also enriched in P42M mice. Ensheathment of neurons was an enriched term shared between P42M and P42F mice (Fig. 1C). Downregulated terms and pathways related to ribosomes, protein translation and protein targeting were only found in P30M mice (Fig. 1D; Supplementary Fig. 1K). In a comparison of enriched GO terms between groups, more common GO terms were found to be shared between P30M and P30F mice than between other groups (Supplementary Fig. 1I). Most of these terms were related to the inflammatory/immune response and the regulation of PI3K signalling, while terms related to oligodendrocytes were shared between all groups except P42F mice (Supplementary Fig. 1I). Taken together, the immune response appeared to be commonly enriched in P30 mice from different groups, whereas oligodendrocyte differentiation was a common transcriptomic change in all groups.

As PTEN has been extensively implicated in ASD, we compared the DEGs of the mice in this study with the weighted gene co-expression network of human brain development^45^. We found that modules 8, 13, 15 and 18, which correspond to the negative regulation of neuron differentiation, synaptic transmission, the response to viruses and the defense response, respectively, were significantly enriched (Supplementary Fig. 1L,M)^45^. The following nine genes were shared by the neuron/synaptic transmission and virus/defense response modules: *LGALS3BP*, *SLC1A3*, *CYBRD1*, *TLN1*, *LPAR1*, *ATP1A2*, *SLCO2B1*, *CLDN11* and *B2M* (Supplementary Table ST1). Only module 17 was enriched with downregulated genes. This module was also related to synaptic transmission, with the following 12 DEGs shared between groups: *TBRG1*, *GPRASP1*, *BEX4*, *SSTR1*, *YPEL4*, *ARPP21*, *SNCA*, *CDH9*, *OPRK1*, *NCALD*, *ZNF365* and *ABI3BP*. Of note, similar transcriptional alterations have been observed in the *Pten*^*m3m4*^ mutant mouse model^51^.

### Proteomic analysis of the somatosensory cortex reveals perturbations of dendritic spine development, keratinisation and hamartoma signatures

**Figure 2:**
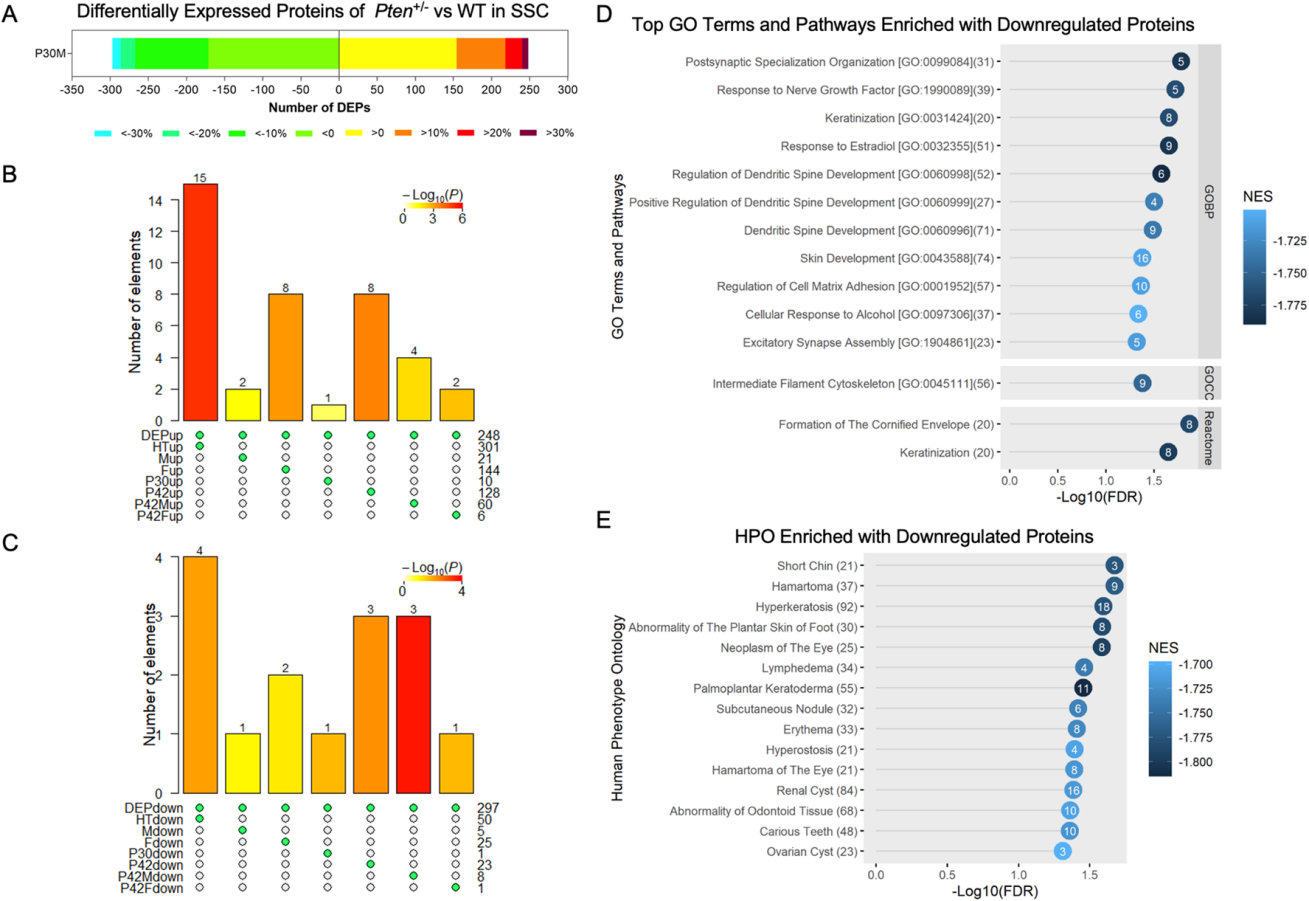
Proteomic analysis of somatosensory cortex of *Pten*^+/−^ mice. (**A**) Number of differentially expressed proteins (DEPs; false discovery rate < 0.05) stratified by the percentage fold-change in the somatosensory cortex of male *Pten*^+/−^ mice (n = 3) and littermate controls (WT) (n = 3) at P30. (**B** & **C**) Upset plots show the number of overlapping differentially expressed genes (DEGs) and DEPs in somatosensory cortices, where DEPup indicates upregulated proteins and DEPdown indicates downregulated proteins. Other groups are DEGs. (**D**) Gene Ontology terms and pathways and (**E**) Human Phenotype Ontology terms significantly enriched with three or more proteins. Number in the rightmost blanket on the left indicates the total number of genes in the term/pathway. Number in the circle indicates the number of proteins involved in the enrichment.

Proteomics analysis was performed using the SSCs of the same mice used for RNA-seq analysis. The P30M group was chosen, because a significant increase in spine density has been observed in *Tsc2*-null mice with mTOR hyperactivation^46^. Of the proteins with human orthologs, 545 proteins were found with Benjamini–Hochberg-corrected false discovery rates (FDRs) < 0.05, consisting of 248 upregulated and 297 downregulated proteins (Fig. 2A). By integrating the differentially expressed proteins (DEPs) and DEGs of all pooled groups, we found that only 15 were upregulated and 4 were downregulated consistently (Fig. 2B,C; Supplementary Fig. 2A,B; and Supplementary Table ST2a, ST2b). Intriguingly, by querying the Brain RNA-Seq database^52,53^, we observed three groups of upregulated proteins. One group, including HAVCR2, CRYBB1, C1QC, MPEG1 and TLN1, was mainly expressed in microglia and macrophages. The second group, including GSN, CERS2 and S100A6, was mainly expressed in oligodendrocytes. The third group was widely expressed in different neural cells and included GRB14 and PLIN3, which are expressed in oligodendrocytes and endothelial cells; CD82, which is expressed in oligodendrocytes, microglia, macrophages and endothelial cells; and BCAN, which is expressed in oligodendrocytes and astrocytes (Supplementary Fig. 2A,B). In contrast, the downregulated protein FXYD6 was mainly expressed in neurons and oligodendrocyte precursor cells (OPCs), and the downregulated proteins ATP2B4 and GPM6A were expressed in ASTs, neurons and OPCs (Supplementary Fig. 2A,B). These results suggest that different neural cells may be affected differently by the heterozygous loss of *Pten*.

Next, we observed that the GO and canonical pathways were only enriched with downregulated DEPs, and postsynaptic specialisation organisation and dendritic spine development were among the top neural-related pathways in the *Pt*en^+/−^ SSC proteome (Fig. 2D). Interestingly, keratinisation, which is one of the clinical diagnostic criteria for Cowden syndrome, was also perturbed, but its function in brain development is unknown. To understand the relationship between the DEPs and enriched pathways, we filtered out proteins with an FDR > 0.05, and then plotted the DEPs against their associated pathways (Supplementary Fig. 2C). We observed that PTEN, RELN, PSEN1, TIAM1 and PPFIA2 shared associations with postsynaptic and dendritic spine development, whereas DSC1, KRT2, KRT15, KRT1 and KRT77 were associated with keratinisation and skin development (Supplementary Table ST2c). NRIP1 inactivation has been shown to be involved in cognitive impairment in mice^54^ and de novo mutations in *KAT5*^55^ and *WASF1*^56^ are associated with cerebral malformations, seizures and developmental delay. GJA1 is predominantly located at astrocytic gap junctions. It has been shown to modulate synaptic plasticity and is predicted to be a driver of Alzheimer’s disease^57^. Taken together, these results indicate that downregulated proteins in the *Pten*^+/−^ SSC may affect cognitive function by changing synaptic development. However, their effect on keratinisation requires further investigation.

To understand how these DEPs are associated with known phenotypes, we performed enrichment analysis using the Human Phenotype Ontology database (Fig. 2E, Supplementary Fig. 2D; Supplementary Table ST2d). Benign tumour-like growth is a diagnostic feature of PHTS. Consistent with this phenotype, the DEPs we identified were enriched with gene sets related to aberrant hypertrophic growth. Hamartoma and neoplasm of the eye were associated with KRT1, VHL and SLC25A11. Various skin abnormalities, such as hyperkeratosis, abnormality of the plantar skin of the foot, palmoplantar keratoderma, subcutaneous nodules and erythema, were associated with GJA1, KRT1, KRT2, PSEN1 and INSR. Hyperostosis, abnormalities of odontoid tissue and carious teeth are problems related to bone or hard tissues, and these were associated with common proteins, such as GJA1 and SLC24A4. Collectively, proteins downregulated in the cortex of the *Pten*^+/−^ mouse model were associated with PHTS pathologies in multiple tissues.

### Transcriptomic analysis of *Pten*-haploinsufficient primary neural cells reveals a major perturbation of immediate early genes

To understand the effect of *Pten* haploinsufficiency on three important neural cell types, we first investigated the PI3K pathway in primary cultures of NPCs, ASTs and PCNs. NPCs at E12-13 were chosen because they are neural lineage cells that have multiple possible cell fate determination pathways. Moreover, the level of PTEN in *Pten*^+/−^ NPCs was reduced by > 70%, but the levels of p-AKT and p-S6 were not significantly elevated. (Supplementary Fig. 3A,B). These results suggest that the remaining wild-type allele in *Pten*^+*/*−^ NPCs is sufficient to suppress aberrant PI3K hyperactivation. The level of p-AKT, but not p-S6, was significantly increased in primary ASTs from the frontal cortices of *Pten*^+/−^ mice, concurrent with a greater than 70% reduction in PTEN protein levels (Supplementary Fig. 3C,D). These results suggest that Akt activity may be more sensitive to *Pten* depletion in primary astrocytes. Finally, for PCNs from the frontal cortices of E15.5 to E16.5 mouse embryos, *Pten* heterozygosity did not alter p-AKT or p-S6 levels, but PTEN levels were reduced by half as expected (Supplementary Fig. 3E,F). These findings suggest that PI3K signalling is not affected by *Pten* depletion in PCNs. Collectively, only very subtle or cell-type-specific effects on PI3K signalling were observed in *Pten*^+/−^ neural cells.

**Figure 3:**
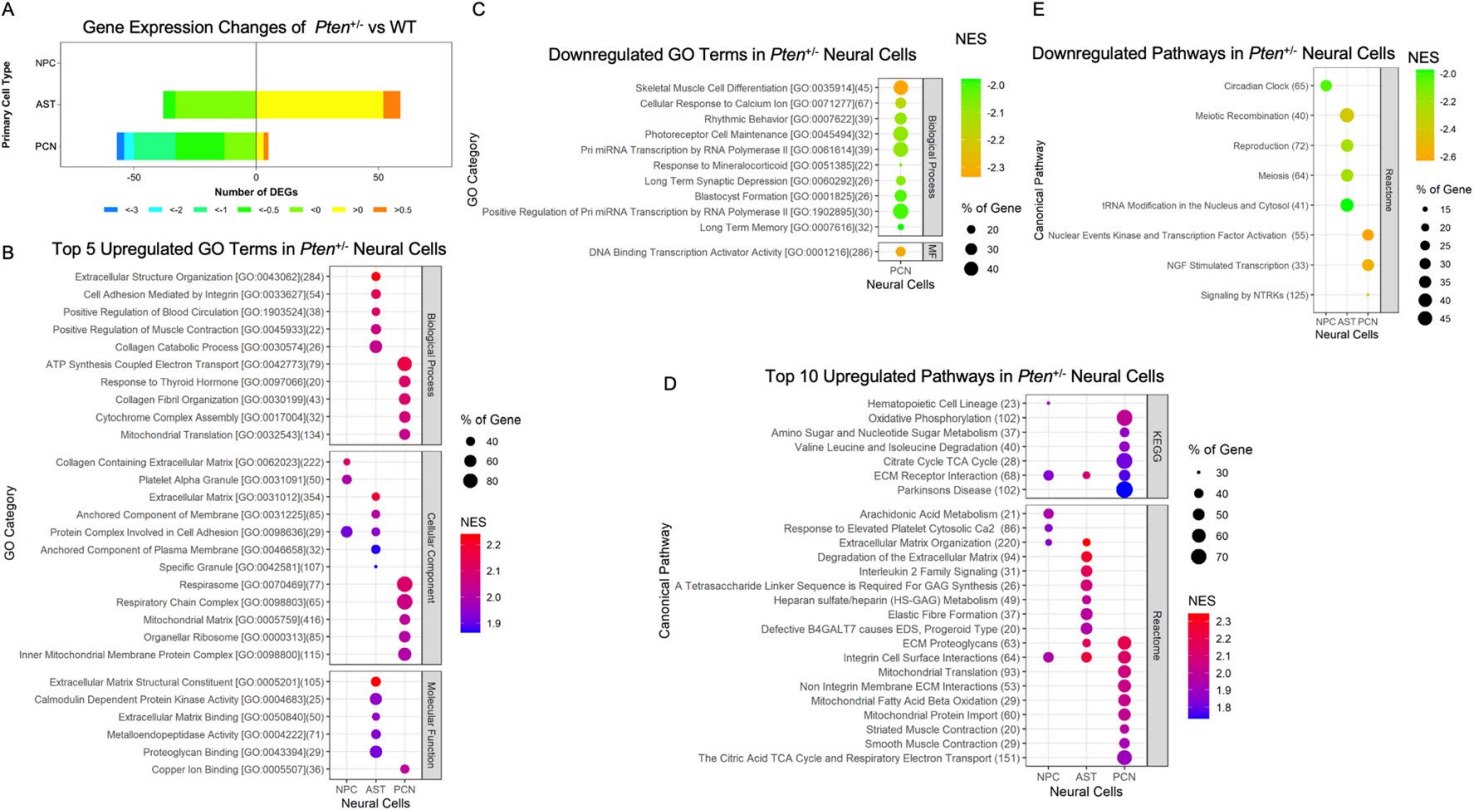
Transcriptomic analysis of *Pten*^+/−^ primary neural cells reveals major perturbation of immediate early genes in neurons. (**A**) The differentially expressed DEGs) are stratified by the percentage fold-change in primary cultures of neural progenitor cells (NPCs), astrocytes (ASTs) and primary cortical neurons (PCNs) from *Pten*^+*/*−^ (n = 3) and wild-type littermate control (WT) (n = 3) mice. (**B**) The five most significantly upregulated Gene Ontology (GO) terms (false discovery rate [FDR] < 0.05) enriched in *Pten*^+/−^ primary neural cells. (**C**) Downregulated GO terms (FDR < 0.05) enriched in *Pten*^+/−^ primary neural cells. No enriched GO terms in NPC and AST. (**D**) The 10 most significantly upregulated canonical pathways (FDR < 0.05) enriched in *Pten*^+/−^ primary neural cells. (**E**) Downregulated canonical pathways (FDR < 0.05) enriched in *Pten*^+/−^ primary neural cells.

Next, we investigated the transcriptomic changes in neural cells from *Pten*^+/−^ mice in comparison with those from their littermate controls. Overall, NPCs were the least affected cells, with no DEGs identified. ASTs had 59 upregulated and 38 downregulated genes. Interestingly, PCN had only 5 upregulated genes, but 57 downregulated genes (Fig. 3A; Supplementary Fig. 3G–I). Notably, most of the downregulated DEGs in PCNs from *Pten*^+/−^ mice were immediate-early response genes (IEGs), namely, *Btg2*, *Dusp1*, *Erg1*, *Fos*, *Fosb*, *Ier2*, *Jun*, *Junb*, *Maff*, *Nr4a1*, *Nr4a2*, *Npas4*, *Ptp4a1* and *Trib1* (GSE190879). IEGs are known to be involved in synaptic plasticity and memory formation and have been implicated in psychiatric disorders^58–61^. It is paradoxical that these IEGs were not detected as DEGs in the SSC from *Pten*^+/−^ mice (data not shown).

Enrichment analysis of GO gene sets and canonical pathways showed that NPCs had the least number of enriched terms. Only a few cellular components and pathways related to the extracellular matrix (ECM), platelet granule and cell adhesion were upregulated, and the circadian clock was the only pathway that was suppressed (Fig. 3B,D,E). More enriched GO terms and pathways were observed in ASTs (Fig. 3B,D,E; Supplementary Fig. 3H). Some of these were similar to those observed in NPCs, such as upregulated ECM-related and cell adhesion pathways. Pathways uniquely enriched in ASTs included blood circulation, muscle contraction, protein kinase and metalloendopeptidase activity, interleukin 2 family signalling and heparin sulphate/heparin metabolism, which were upregulated, and pathways related to meiosis regulation, which were downregulated. Finally, PCNs had many uniquely enriched gene sets (Fig. 3B–E; Supplementary Fig. 3I). The upregulated gene sets in PCNs were mostly related to mitochondrial function and energy production, such as ATP synthesis-coupled electron transport, the respirasome, sugar metabolism and the tricarboxylic acid cycle. Downregulated gene sets included functions such as pre-microRNA transcription by RNA polymerase II, DNA-binding transcription activator activity, long-term synaptic depression and long-term memory. Taken together, these data suggest that the heterozygous loss of *Pten* induces undetectable to very low gene expression and very mild functional perturbations in different neural cells.

### Transcriptomic analysis of *Pten*-knockout primary neural cells uncovers neural-cell-specific signatures

**Figure 4:**
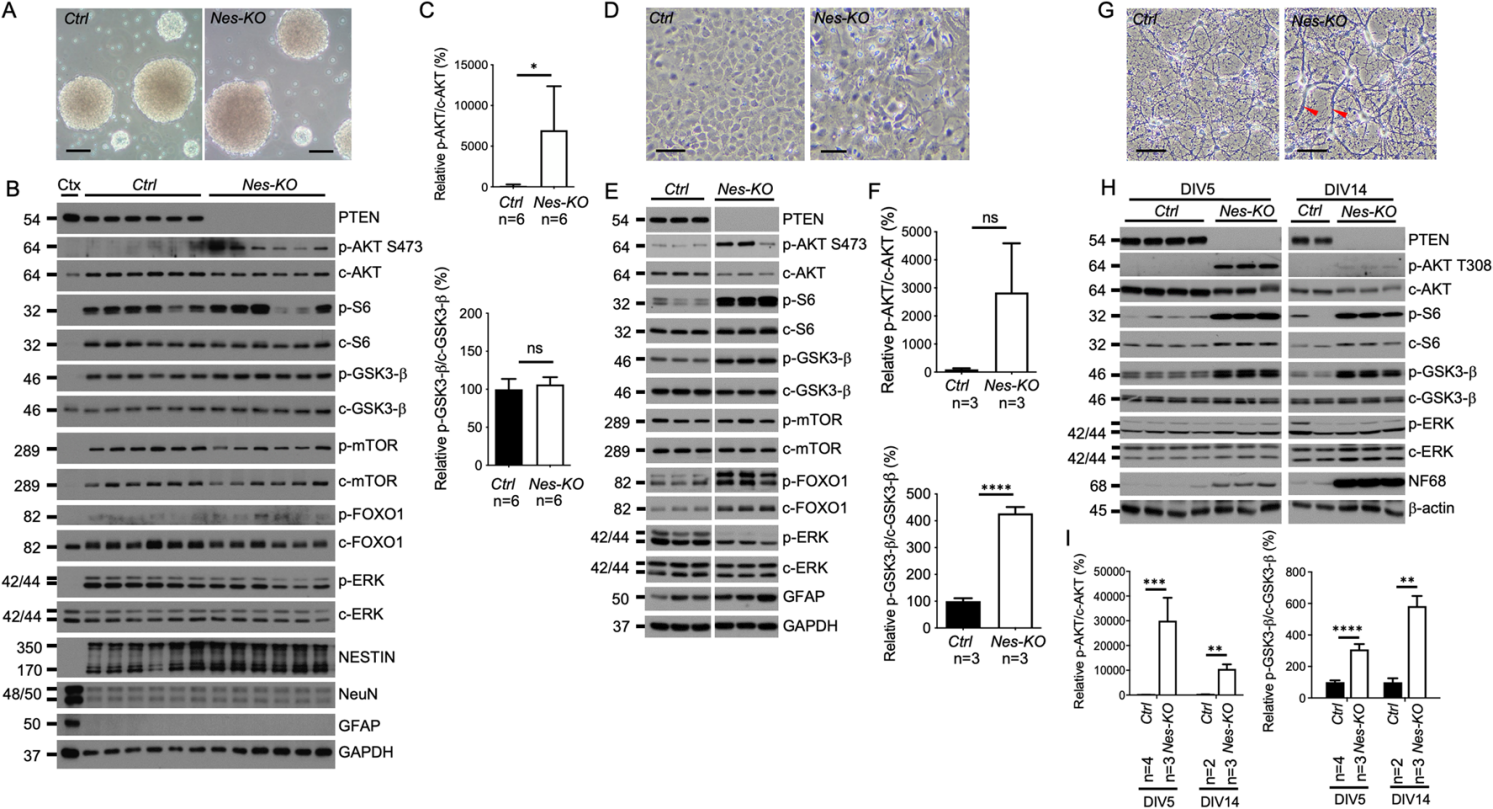
Aberrant PI3-K signalling in *Pten*-knockout neural cells. Representative bright-field images of primary cultures of (**A**) neural progenitor cells (NPCs), (**D**) astrocytes (ASTs) and (**G**) neurons at DIV14. Scale bar = 0.1 mm. Red arrowheads, hypertrophic neurites. Immunoblotting of the indicated proteins in (**B**) NPCs, (**E**) ASTs and (**H**) neurons of *Pten*-knockout (*Nes-KO*) and littermate control (*Ctrl*) mice. Cortical lysate from a wild-type mouse (Ctx) was used as a control. The specific phosphorylation of p-AKT and p-GSK3-β relative to control (*Ctrl*) are shown in (**C**) NPCs, (**F**) ASTs and (**I**) PCN. An unpaired Student’s t-test was used for statistical analysis with the indicated p-values: * < 0.05, ** < 0.01, *** < 0.001 and **** < 0.0001.

The heterozygous loss of *Pten* resulted in subtle transcriptional changes. We hypothesised that the complete knockout of *Pten* would unambiguously reveal disease-associated DEGs. Nestin (*Nes*)-*Cre* transgenic mice were crossed with *Pten*^fl/fl^ mice to generate complete *Pten* knockout (*Nes-KO*) mice. The brains of *Nes-KO* embryos were visually distinguishable from littermate controls at E16.5 and were more strikingly different at P0 (Supplementary Fig. 4). Thus, *Pten* is required for correct brain development at the commencement of neurogenesis^62^. NPCs, ASTs and PCNs were cultured as aforementioned (Fig. 4A,D,G). Western blotting analysis showed that p-AKT was upregulated in all neural cells relative to control (Fig. 4B,C,E,F,H,I). Interestingly, other PTEN-related signalling molecules, such as p-S6, p-mTOR, p-GSK3β, p-FOXO1 and p-ERK, were not significantly altered in NPCs (Fig. 4B,C). In contrast, levels of p-S6 and p-GSK3β were elevated in ASTs and PCNs, which was consistent with the results of previous studies^63,64^. These data suggest that different neural cell types have distinct responses to *Pten* knockout.

Furthermore, the levels of the early neuronal marker NeuN and the astrocyte marker GFAP remained low in *Nes-KO* NPCs (Fig. 4B). This indicates that the loss of *Pten* did not affect the neural differentiation of NPCs obtained at E12-13. In ASTs, we observed elevated expression levels of GFAP in *Nes-KO* cells (Fig. 4E). Increased GFAP levels may suggest reactive astrogliosis, which is commonly observed in hypertrophic astrocytes^17,65^. Therefore, the complete knockout of *Pten* in ASTs may lead to the hyperactivation of the PI3K pathway and the promotion of astrogliosis. In PCNs, we noted that the levels of NF68, a major constituent of the axonal cytoskeleton^66,67^, were markedly increased in the *Nes-KO* group when the cultures were matured from 5 Days In Vitro (DIV5) to DIV14 (Fig. 4H). Moreover, hypertrophic neurons with thick neurites were observed at DIV14 (Fig. 4G). Thus, the complete depletion of *Pten* in neurons leads to PI3K activation and is associated with hypertrophic axonal growth.

**Figure 5:**
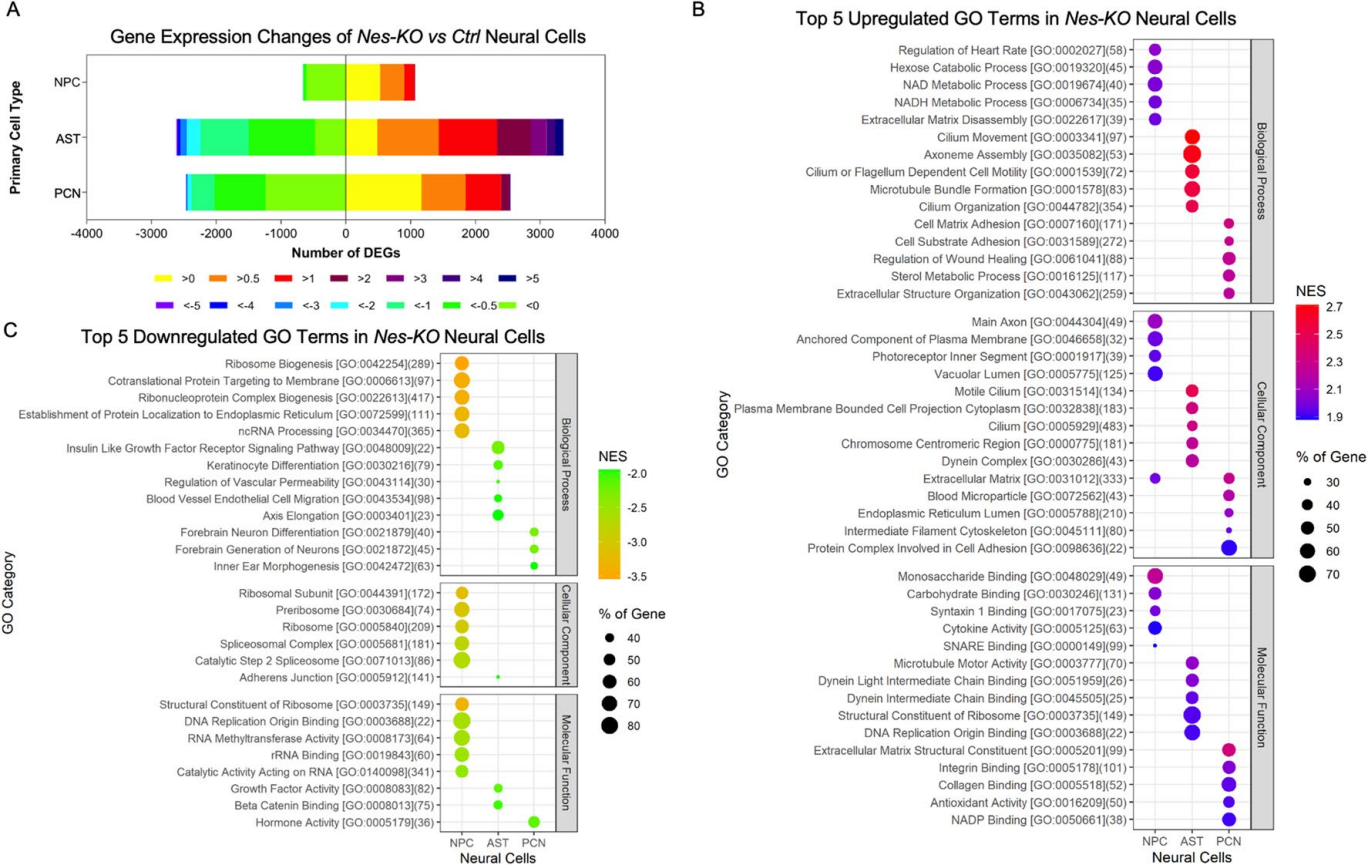
Transcriptomic analysis of *Pten*-knockout primary neural cells uncovers neural cell-specific signatures. (**A**) Differentially expressed genes (DEGs) in primary cultures of neural progenitor cells (NPCs)(n = 3), astrocytes (ASTs)(n = 3) and primary cortical neurons (PCNs)(n = 3) from *Pten*-knockout (*Nes-KO*) mice and littermate controls (*Ctrl*) (n = 3) are stratified by log_2_ fold-change. (**B**) The five most significantly upregulated Gene Ontology (GO) terms (false discovery rate [FDR] < 0.05) enriched in *Nes-KO* primary neural cells. (**C**) The five most significantly downregulated GO terms (FDR < 0.05) enriched in *Nes-KO* primary neural cells.

RNA-seq analysis showed that *Nes-KO* NPCs had the fewest DEGs, with 1,070 upregulated and 662 downregulated protein-coding genes, but only 170 upregulated and 9 downregulated genes, based on a log_2_ fold-change (FC) > 1 (Fig. 5A; Supplementary Fig. 5A–E). *Nes-KO* ASTs had 3359 upregulated and 2615 downregulated genes, of which 1926 upregulated and 1118 downregulated genes had an absolute log_2_FC of >1. Strikingly, the maximal expression differences ranged from log_2_FC − 7.8 to + 12.7. *Nes-KO* PCNs had 2541 upregulated and 2462 downregulated genes, with 694 and 439 of those, respectively, showing an absolute log_2_FC > 1. Collectively, the transcriptomes of *Nes-KO* ASTs showed the greatest perturbations compared with other neural cells after the complete loss of *Pten*.

Gene set enrichment analysis showed that *Nes-KO* NPCs had upregulated genes related to energy metabolic processes, such as hexose catabolic, NAD and NADH metabolic processes; carbohydrate binding and gluconeogenesis (Fig. 5B; Supplementary Fig. 5A,C), and downregulated ribosome-related processes, such as ribosome and ribonucleoprotein complex biogenesis and processes involving the ribosomal subunit, preribosome, ribosome and spliceosomal complex, along with related molecular functions, such as DNA replication and rRNA binding (Fig. 5C; Supplementary Fig. 5B,C). These results indicate that upregulated energy metabolism and downregulated mRNA translation and protein expression are signatures of *Nes-KO* NPCs.

In *Nes-KO* ASTs, components of cilia and ribosomes and mitosis-related pathways, were among the most upregulated pathways (Fig. 5B; Supplementary Fig. 5A,D), whereas relatively few downregulated pathways, such as the insulin-like growth factor-1 receptor signalling pathway, endothelial cell migration, growth factor activity and the JAK-STAT signalling pathway, were identified (Fig. 5C; Supplementary Fig. 5B,D). These results point to upregulated cilial activities, mRNA translation and mitosis as the main signatures of *Nes-KO* ASTs.

In *Nes-KO* PCNs, major changes included upregulated ECM components and cell adhesion, sterol metabolic process, leukocyte transendothelial migration and MET activation of PTK2 signalling (Fig. 5B; Supplementary Fig. 5A,E), and downregulated forebrain neuron generation and differentiation (Fig. 5C; Supplementary Fig. 5B,E). Of note, most of these downregulated genes, such as *Ascl1*^68^, *Lhx6*, *Arx*, *Dlx1*, *Dlx2*, *Dlx5*^69^, *Erbb4*^70^, *Prox1*^71^ and *Foxg1*, are confined to GABAergic neurons or act as determinants of differentiation into inhibitory neurons^72^. At the same time, the levels of some secretory proteins specific to inhibitory neurons, such as RELN, ERBB4, SST and NPY, were decreased (Supplementary Fig. 6). These findings suggest that upregulated genes were involved in multiple functions, whereas downregulated genes implicated in E/I balance were the most notable signatures of *Nes-KO* PCNs.

### Linking *Pten* haploinsufficiency to intelligence, cognitive function and schizophrenia

**Figure 6:**
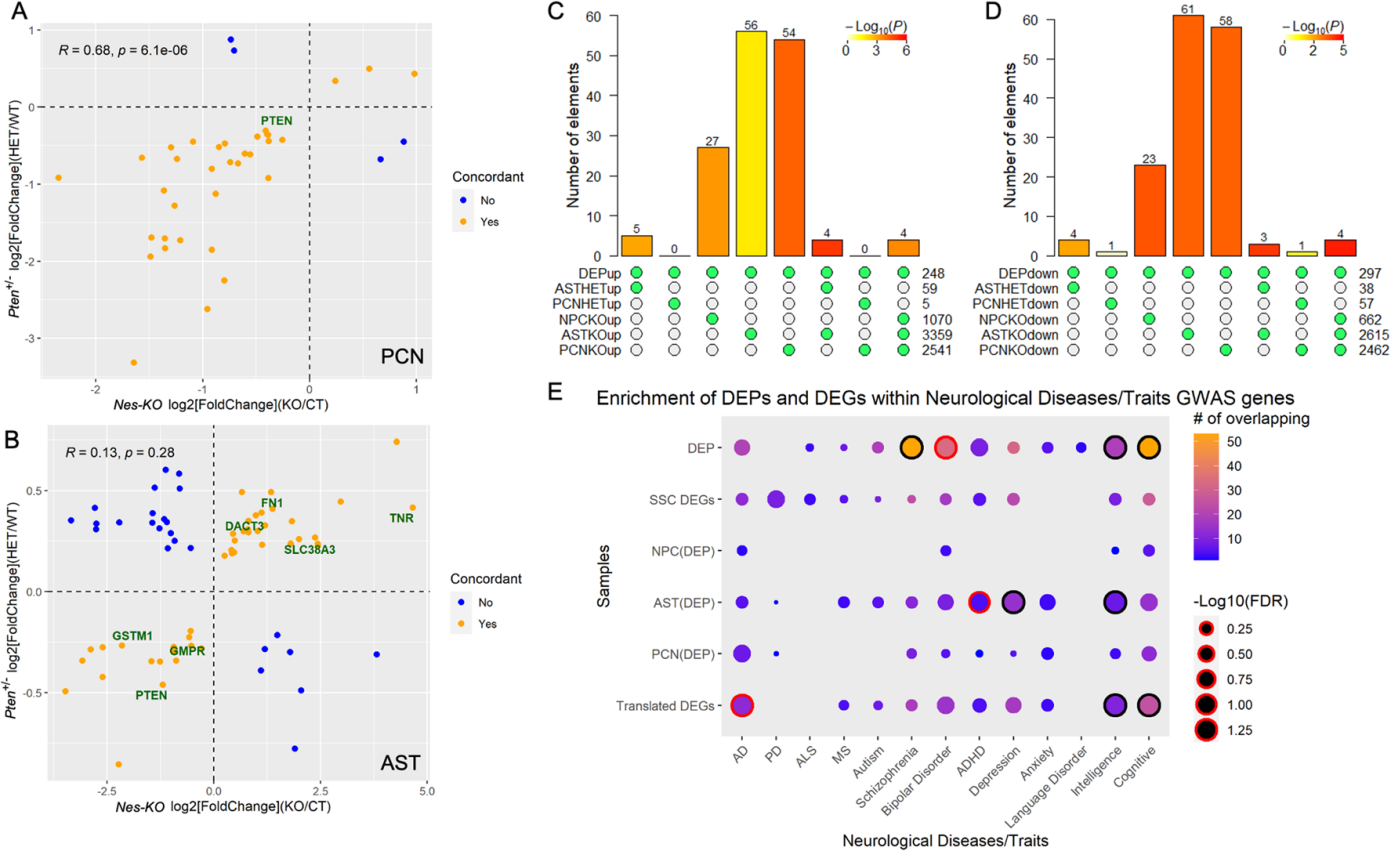
Linking *Pten* haploinsufficiency to intelligence, cognitive function and schizophrenia. Gene expression fold-change dimensionality comparison of common differentially expressed genes (DEGs) between *Pten*^+/−^ and *Nes-KO* (**A**) primary cultures of neurons and (**B**) astrocytes (ASTs). R: Spearman’s correlation coefficient. Overlapping (**C**) upregulated and (**D**) downregulated differentially expressed proteins (DEPs) in the somatosensory cortex of *Pten*^+/−^ mice with DEGs from *Pten*^+/−^ (HET) and *Nes-KO* (KO) neural cells. Statistical analysis of enrichment was performed using SuperExactTest. (**E**) Enrichment of DEPs and DEGs in the somatosensory cortex of *Pten*^+/−^ mice and translated DEGs in neural cells converging with DEPs and all translated DEGs (adding up translated DEGs from all neural cells) within genes identified in GWAS of neurological diseases and traits. Dots enclosed by a black circle represent enrichment with FDR < 0.05. Dots enclosed by a red circle represent enrichment with FDR < 0.1. Statistically significant enrichment was calculated using an FDR-corrected cumulative hypergeometric test.

Next, we explored the potential neurological disorders and traits associated with *Pten* haploinsufficiency. First, the gene expression correlations between heterozygous and homozygous *Pten*-knockout PCNs and ASTs were compared by Spearman’s correlation analysis (Fig. 6A,B). The results showed that the DEGs between PCNs from *Pten*^+/−^ and *Nes-KO* mice were significantly correlated (ρ = 6.1e−06, R = 0.68; Fig. 6A), but this was not observed in ASTs (Fig. 6B). Next, we curated gene lists by integrating DEGs with DEPs from the SSC of P30M *Pten*^+/−^ mice. Of the different neural cells, ASTs from *Nes-KO* mice showed the greatest number of overlapping DEGs and DEPs, followed by PCNs from *Nes-KO* mice (Fig. 6C,D; Supplementary Table ST3a, ST3b). Overall, there was a lack of concordance between PCN DEGs of *Pten*^+/−^ mice and SSC DEPs. This may be due to the differences between neuronal cells in vitro and in vivo. Alternatively, the complete depletion of *Pten* may have highly distorted the gene expression profiles, compared with those in *Pten*^+/−^ mice, such that DEPs may not be detected in the SSC of *Pten*^+/−^ mice.

Finally, to uncover the potential neurological disorders and traits associated with *Pten* haploinsufficiency, we curated gene lists by integrating DEPs from the SSC of *Pten*^+/−^ mice and DEGs from neural cells of *Pten*^+/−^ and *Nes-KO* mice by filtering out genes encoding non-DEPs from the DEGs of neural cells as NPC(DEP), AST(DEP), PCN(DEP) and curating translated DEGs comprising DEGs from all neural cells that overlapped with DEPs (Supplementary Table ST3c). Next, we performed enrichment analysis between the gene lists and the genetic variants associated with neurological disorders and traits obtained from genome-wide association studies (Fig. 6E; Supplementary Table ST3d)^73^. Overall, we found significant enrichment (FDR < 0.05) of both DEPs and translated DEGs in the traits of intelligence and cognitive function, and proteins involved in schizophrenia were significantly enriched in the DEPs (Fig. 6E; Supplementary Table ST3e). Further investigation at the cell-type level showed that AST(DEP) was significantly associated with intelligence and depression.

## Discussion

In the present study, we made the following key observations (see supplementary Table ST4 for a summary). First, the levels of PTEN in *Pten*^+/−^ neural cells fluctuated widely, ranging from 10 to 50%. Second, immediate-early genes were enriched as DEGs in *Pten*^+/−^ PCNs, which implies an important function of PTEN in the regulation of neuronal activity. Unexpectedly, none of these IEGs were found as DEPs in the SSC of *Pten*^+/−^ mice. One explanation for this finding is that the expression levels of IEGs readily change upon neuronal stimulation or depolarisation^74,75^. Indeed, cultured PCNs represent an unstimulated, naïve state. In contrast, the somatosensory region of mice is constantly being stimulated, for example, by the whiskers^37^. Another reason for this finding is that the dynamic fluctuation in PTEN levels may make downstream gene expression and protein functions too transient to be captured. To address this issue, we characterised the effect of homozygous *Pten* knockout on these neural cells. As expected, the number of DEGs and the number of DEGs that overlapped with DEPs were markedly increased in all neural cells. The drawback of knockout models is the generation of clinically irrelevant effects due to non-physiological transcriptomic changes and hyperactivation of the PI3K pathway. Filtering out these irrelevant genes based on DEPs in brain tissues partially addressed this issue.

A previous transcriptomic study explored the whole brain and cortical tissues from a *Pten*^m3m4^ mutant knock-in mouse line^76^. These mutant mice have reduced nuclear PTEN localisation and exhibit behavioural deficits similar to those seen in high-functioning ASD. Similar to our findings, the brains of homozygous *Pten*^*m3m4/m3m4*^ mutant mice contain more DEGs than those of heterozygous *Pten*^*m3m4/*+^ mutant mice. In addition, we observed that *Nes-KO* transcriptomes contained many more DEGs that integrated with the proteomes of the SSC of *Pten*^+/−^ mice. Therefore, analysing neural cells with complete *Pten* knockout enabled us to identify more PHTS-relevant genes based on the proteomic profile of *Pten*^+/−^ mice.

Many IEGs are transcription factors and their expression is regulated by neuronal activity, such that neuronal circuits can be adjusted to meet functional needs. PTEN has been found localised to different cellular compartments. More recently, postsynaptic density-localised PTEN has been shown to interact with the synaptic scaffolding molecule, PSD-95, through a PDZ-binding motif, which is required for N-methyl-D-aspartate (NMDA) receptor-dependent long-term depression^77^. The excitatory neurotransmitter glutamate binds to the NMDA receptor and induces calcium influx and triggers calcium-dependent signalling, leading to changes in the expression of transcription factors, such as FOS and NPAS4^78^. Interestingly, glutamate binds to glutamate receptors to induce protein-synthesis-dependent long-lasting synaptic plasticity by activating mTOR^79^. Therefore, we speculate that PTEN is involved in both IEG expression and mTOR-dependent protein synthesis.

We uncovered pathways in both ASTs and neurons that may explain the neurological disorders observed in PHTS. In *Nes-KO* ASTs, cilium-related activity and components and mitosis-related pathways showed the most enriched perturbations. ASTs possess a single, non-motile primary cilium^80^ that regulates various functions, such as cell division and signal transduction pathways, and plays important roles in sensory functions^81^. In fact, PTEN regulates cilial turnover by controlling dishevelled phosphorylation^82^. The dysregulation of ciliogenesis causes behavioural and cognitive defects^83^ and brain malformations^84^. Furthermore, *Nes-KO* neurons exhibited decreased forebrain neuron generation and differentiation, which may lead to disruption of the E/I balance in the brain. This included the downregulation of genes that are required for differentiation into GABAergic neurons. Similarly, conditional *Pten* knockout driven by *Nkx2.1-Cre* results in the preferential loss of SST-positive interneurons^31^. However, whether changes in cilia and interneurons are present in PHTS patients remains to be confirmed.

Through our DEP and translated DEGs lists, we uncovered associations between PHTS and schizophrenia, intelligence and cognitive function. In fact, a pathogenic *PTEN* mutation (Gln219*) has been reported in a patient with Cowden syndrome complicated by schizophrenia^85^. There were more than 50 proteins in our list of DEPs that matched with schizophrenia genome-wide association study (GWAS) data, and the genes encoding 18 of these were identified as DEGs in the transcriptomic analysis. One of these proteins was RELN, which is preferentially expressed in GABAergic interneurons of prefrontal cortices, temporal cortex and hippocampus^86^. The protein level of RELN was downregulated by 17%, but the transcript level was downregulated by 67% (GSE190879). Other PHTS cognitive manifestations, such as intellectual abilities^87^, were also enriched in our DEP/DEGs list. Furthermore, it is worth noting that AST(DEP) was significantly associated with depression and intelligence, which may provide a new direction for research on the function of PTEN in astrocytes.

Our study has several limitations. While the *Pten*^+/−^ mouse model recapitulates most of the pathological features of PHTS, potential species-specific differences may have confounded the results. Moreover, we only analysed the SSC region. Other neuroanatomical regions may have molecular changes associated with the pathological features of PHTS. Another caveat is the possibility that neural cell types other than NPC, AST and PCN may play critical roles in PHTS. For instance, microglia have been implicated as key regulators of neuroinflammation and they play a role in neurodevelopmental and neurodegenerative disorders^88^. Future studies should explore the use of single-cell RNA-seq analyses of fresh brain tissues from *Pten*^+/−^ mice.

In summary, we identified multiple pathways and genes that are perturbed in *Pten*-deficient cells and tissues. Further, by integrating transcriptomic and proteomic profiles, we identified several neurological diseases and traits that may be relevant to the neuropathogenesis of PHTS.

## Supplementary Information

Supplementary Information 1.

Supplementary Information 2.

Supplementary Information 3.

Supplementary Information 4.

Supplementary Information 5.

Supplementary Information 6.

Supplementary Information 7.

Supplementary Information 8.

Supplementary Information 9.

Supplementary Information 10.

Supplementary Information 11.

Supplementary Information 12.

Supplementary Information 13.

## Data Availability

Transcriptomic data: GEO accession GSE190879. The mass spectrometry proteomics data have been deposited to the ProteomeXchange Consortium via PRIDE, under the identifier PXD030573.

## Abbreviations

AST: Astrocytes
ASD: Autism spectrum disorders
DEGs: Differential expressed genes
DEPs: Differentially expressed proteins
DCX: Doublecortin
E/I: Excitatory/inhibitory
ECM: Extracellular matrix
FDR: False discovery rate
GO: Gene Ontology
HPO: Human phenotype ontology
IEGs: Immediate-early response genes
mTOR: Target of rapamycin
NMDA: N-methyl-d-aspartate
NSE: Neuron-specific enolase
NPC: Neuroprogenitor cell
OPC: Oligodendrocyte precursor cell
PI3K: Phosphoinositide 3-kinase
PCN: Primary cortical neurons
PHTS: PTEN hamartoma tumour syndrome
SSC: Somatosensory cortex
SST: Somatostatin
SVZ: Subventricular zone
