# Supplementary material for: Neuropathological signatures revealed by transcriptomic and proteomic analysis in Pten-deficient mouse models
Source: Sci Rep. 2023 Apr 25;13:6763. doi: 10.1038/s41598-023-33869-7 (PMC10130134; doi:10.1038/s41598-023-33869-7)

**Supplementary Information File**

**Neuropathological signatures revealed by transcriptomic and proteomic analysis in *Pten*-deficient mouse models**

Stanley K.K. Cheung,^1^ Jacinda Kwok,^1,#^ Penelope M.Y. Or,^1^ Chi Wai Wong,^1,@^ Bo Feng,^1^ Kwong Wai Choy,^2^ Raymond C.C. Chang^,3^ J. Peter H. Burbach,^4^ Alfred S.L. Cheng^1^ and Andrew M. Chan^1,5^*

**Primary cultures**

All cell culture reagents were purchased from Thermo Fisher Scientific unless otherwise indicated. Primary NPCs were established from the forebrains of embryonic day 11.5 (E11.5) – E12.5 mouse brains, as described previously.(1) Acutely dissociated single cells were cultured in F-12/Dulbecco’s modified Eagle’s medium (DMEM), N2 supplement, B27 supplement, 20 ng/ml mouse epidermal growth factor, and 20 ng/ml mouse basic fibroblast growth factor. Neurospheres were passaged once and seeded at a density of 5 × 10^5^ cells per 100-mm plate and collected on the top of cell strainer (40μm, Corning). To obtain PCAs, the cortical regions were microdissected from mouse brains at postnatal days 0–2 (P0–2). Cells were plated on 60-mm culture dishes coated with 10 μg/ml poly-D-lysine (Sigma) and maintained in DMEM plus 10% fetal bovine serum (FBS) and 1x antimycotic. Cells were harvested at confluence.(2) PCNs were established from cortices microdissected from E15.5 embryos, as described previously.(3) Acutely dissociated cells from a single brain were plated at 4.5x10^6^ cells per dish onto two 60-mm culture dishes coated with poly-D-lysine and incubated with neurobasal medium supplemented with 1× B27, 1× penicillin/streptomycin, 1× antimycotic, and 0.5 mM L-glutamine. AraC (1 μM) was added to the cultures on the first day to eliminate microglial contamination. Cultures were collected on either day in vitro (DIV) 5 or 14.

**Western blotting analysis**

The cells and SSC were solubilized in RIPA buffer composed of 50 mM Tris-HCl pH 7.5, 1% Triton X-100, 0.5% sodium deoxycholate, 0.1% SDS, 150 mM NaCl, 10 mM MgCl2, and protease and phosphatase inhibitors. Approximately 5 or 10 µg of each cell lysate was resolved on 10% SDS-PAGE gels, transferred onto nitrocellulose membranes, and probed with selected primary antibodies (see below for a full list). The bound antibodies were detected with horseradish peroxidase (HRP)-conjugated secondary antibodies as appropriate. To determine the protein expression levels, the blots were visualized using the standard ECL method and exposed to X-ray films. The band intensities were quantified using the GS-900 Calibrated Densitometer System (Bio-Rad).

**RNA-seq analysis**

Cleaned sequencing reads from groups of *Pten*^+/-^ or Nes-KO mice, together with their littermate controls, were analyzed separately using the mouse genome version 10 (mm10) as the reference genome. Reads were aligned using HISAT2.(4) The mapped reads were quantified using featureCounts(5). Raw count data were tested for differential expression using the DEseq2 package.(6) Raw data and normalized counts are available as Gene Expression Omnibus series GSE190879. Biotype of genes were mapped using biomaRT.(7) Only protein coding genes and genes with count over 100 were selected for all downstream analyses. Gene Ontology (GO), canonical pathways and human phenotype ontology (HPO) enrichment analyses were performed using the Gene Set Enrichment Analysis (GSEA) v4.1.0 (Broad Institute)(8, 9) with Molecular Signature Database 7.2 was used to analyze the full transcriptome set.(10) Statistical significances between sets of DEGs and DEPs were tested using SuperExactTest package.(11) An adjusted p-value or FDR of <0.05 was considered statistically significant. Redundant GO terms were removed by Revigo (12). Volcano plots were made using EnhancedVolcano (version 1.16.0)(downloaded from <https://github.com/kevinblighe/>EnhancedVolcano).

**Proteomic analysis**

Snap-frozen SSCs were lysed on ice with 10 times volume of RIPA buffer supplemented with cocktail of protease and phosphatase inhibitors by passing through 25-G needle for 10 times. After centrifuged at 16,000x for 10 minutes at 4^o^C, 100 µg total soluble proteins were analysis. For iTRAQ analysis, the samples were diluted in buffer with EDTA and without SDS. Final concentration of 10mM DTT was added to reduce disulfide bonds in proteins, free cysteine thiol group was blocked by 50mM IAM (2-iodoacetamide) . Protein solutions were digested with trypsin at 1:20 (trypsin:total protein) for 4 hours at 37^o^C. After desalting, peptide samples were freeze-dried, reconstituted in 0.5M triethylammonium bicarbonate (TEAB), labeled with different iTRAQ tags and fractionated by Shimadzu LC-20AB liquid phase system using 5μm 4.6x250mm Gemini C18 column. The dried peptide samples were reconstituted with mobile phase A (5% ACN, pH 9.8) and injected with flow rate of 1mL/min in gradients of 5% mobile phase B (95% ACN, pH 9.8) for 10 minutes, 5% to 35% B for 40 minutes, 35% to 95% B for 1 minute, 100% B for 3 minutes, finished with 5% B for 10 minutes.

Eluted samples were monitored at 214nM at fraction per minute and combined according to chromatographic elution peak map to obtained 20 fractions. Freeze-dried samples were reconstituted with mobile phase C (2% ACN, 0.1% formic acid(FA)) and separated in Thermo UltiMate 3000 UHPLC using a self-packed C18 column (75μm internal diameter, 3μm column size, 25cm column length), at 300nL/min in gradients of 5% mobile phase D (98% ACN, 0.1% FA) for 5 minutes, linear increasing B from 5% to 25% from 5 to 45 minutes, increasing B from 25% to 35% from 45 to 50 minutes, 35% to 80% of B from 50 to 52 minutes, 80% of B from 52 to 54 minutes, finished with 5% B from 54 to 60 minutes. The nanoliter liquid phase separation end was directly connected to tandem mass spectrometer Q-Exactive HF X for dataacquisition via ionization by a nanoESI source. Ion source voltage was set to 1.9kV, MS1 scanning range was 350-1,500 m/z with resolution of 60,000, MS2 starting m/z was fixed at 100 with resolution of 15,000.

The ion screening conditions for MS2 fragmentation was charge from 2+ to 6+ with top 20 parent ions with peak intensity over 10,000. The ion fragmentation mode was HCD and the fragment ions were detected in Orbitrap with dynamic exclusion time of 30 seconds. The automated gain control (AGC) target was set to MS1 at 3E6 and MS2 at 1E5. The raw MS/MS data was converted into MGF format which was searched against the local Mascot database server. An automatic software IQuant was used for quantification of proteins.(13) From the p-values obtained, false discovery rate (FDR) with (Benjamini–Hochberg correction) were calculated and less than 0.05 were considered as significant. Preranked protein list were generated by multiplying –log_10_(FDR) and log(fold-change). Enrichment analysis was performed using GSEA as aforementioned. Heatmaps were made using R (version 4.2.2) for GOBP and HPO enrichment analyses from GSEA (version 4.1.0). The heatmaps showed the proteins involved in the significant enrichment with FDR of <0.05. Fold change is the mean ratio of protein levels of HT over WT. Volcano plots were made using EnhancedVolcano (version 1.16.0)(downloaded from <https://github.com/kevinblighe/>EnhancedVolcano).

**Antibody list**

Actin (13E5, #4970), p-AKT S473 (587F11, #4051), p-AKT T308 (#9275), c-AKT (#9272), ERBB4 (111B2, #4795), p-ERK (E10, #9106), c-ERK (#9102), GAPDH (D16H11, #5174), GFAP (GA5, #3670), p-GSK-3β (D85E12, #5558), c-GSK-3β (D5C5Z, #12456), Horseradish Peroxidase (HRP)-conjugated anti-mouse IgG (#7076), HRP-conjugated anti-rabbit IgG (#7074), p-FOXO1 (#9464), c-FOXO1 (C29H4, #2880), NPY (D7Y5A, #11976), SYP (D35E4, #5461), p-mTOR (D9C2, #5536), c-mTOR (7C10, #2983), PTEN (138G6, #9559), p-S6 (D57.2.2E, #4858), and c-S6 (54D2, #2317), were purchased from Cell Signaling Inc. Antibodies against NEUN (A60, #MAB377), SST (YC7, #MAB354) and vGLUT (3C10.2, #MAB5502) were from Millipore. Antibody against NESTIN (Rat-401, #ab11306), NF68 (DA2, #ab72995), RELN (G10, #ab78540), and Horseradish Peroxidase (HRP)-conjugated anti-chicken IgY (#ab6877) were purchased from Abcam. Antibodies against ARX (#AV36825), DLX2 (#AB5726), DLX6 (#AV35764) and Gad65/67 (#G5163) were purchased from Sigma-Aldrich.

**Primer list**

| **Gene** | **Forward, 5'-->3'** | |  |  | **Reverse, 5'-->3'** | |  |
| --- | --- | --- | --- | --- | --- | --- | --- |
| *Actb* | GCTATGTTGCTCTAGACTTCG | | | | GGATTCCATACCCAAGAAGG | | |
| **For neural progenitor cells** | | |  |  |  |  |  |
| *Upregulated* | |  |  |  |  |  |  |
| *Ank3* | TTAGTTGGACAGAGCTGGC | | |  | TCTTTCCGTCTCTGGTCAC | | |
| *Ass1* | TCACCTGCTATTCACTGGC | | |  | GATTCCGTGTTGCTTTGCA | | |
| *Celf4* | TGCAGACACTGACAAGGAG | | |  | ATCAGTGCCTGAGCATAGG | | |
| *Clstn3* | TGTGGCTTACATGAACACTC | | |  | CTAAAGCACTTGACAGCGG | | |
| *Fabp3* | TATCCCCGTTCTTCTCGATG | | |  | CCTTTGTCGGTACCTGGAAG | | |
| *Fgfbp3* | TTCGCTCTACTCCAATAGGG | | |  | AAGGAGCATCCTAGGATCG | | |
| *Kcnq2* | CTGCCTTGTGCTTTCTGTG | | |  | CACGATTTCCAAGATGTAGAGG | | |
| *Myt1l* | CTTCTCATCGAAGCCTTTCTG | | |  | CTTCCTTGTCCTCTTTGCTC | | |
| *Pcdha13* | GCAGGTAGAACATTTGAAAGAG | | | | TGGATACTGTTGGCCACTG | | |
| *Prune2* | ATCTAAAGGAGCTGTCAGACG | | | | CGTGAAGCAGGTAGTCCTC | | |
| *Slc16a3* | AGTTCTTGGTGAAAGCAGC | | |  | CAAACTCATGCATAAGCTCCT | | |
| *Slc4a10* | CTCTACTGTGCATGCATGTC | | |  | GATTGCACTTATACGACCTTCAG | | |
| *Sspo* | CTGCCATAATTGCACATGC | | |  | CTTACCACAGACAGCTGAG | | |
| *Downregulated* | |  |  |  |  |  |  |
| *Thbs1* | GACCCTAACTGGGTTGTCC | | |  | CATCATAACCTACAGCAAGTCC | | |
|  |  |  |  |  |  |  |  |
| **For primary cortical astrocytes** | | |  |  |  |  |  |
| *Upregulated* | |  |  |  |  |  |  |
| *Atp8a1* | AGGTTCCTCTATTCTCAGTTCC | | | | CACATCAGGAATTTGCTGGAG | | |
| *Ccdc88c* | GTCAACAATGATGTCAACCTG | | |  | GAGAACTTCCTGGTAGTAGGT | | |
| *Fabp7* | TGACAGAAACTGTAAGTCTGTG | | | | TTTGTTTCTTTGCCATCCCA | | |
| *Fbxo40* | TCCACAATCAGATCTGGCAG | | |  | CTCAGACATGGAGGTGACG | | |
| *Fezf2* | AGGTGCTGAAGGAGAACTC | | |  | GAGCATTGAACACCTTGCC | | |
| *Gabra4* | TTTCTGTCCAGAAGGTACCC | | |  | TTCGTTTAAACAAGCCGCC | | |
| *Gabrb1* | TGGACTACGGATCACAACC | | |  | TTCAATCTCCAGGGTGCAG | | |
| *Grid2* | GCAAGCTTGTCCTGTATCAG | | |  | AACTCCACCCTTCTTGATGG | | |
| *Kcnj10* | ACCTTCGAGCCAAGATGAC | | |  | GTCTTTCGTGAGGACCCTC | | |
| *Nrxn1* | TTTAGCACTGTCCAGAAGG | | |  | TTCCTTGGTGTATGTGCAG | | |
| *Slc6a1* | TGAACTCTTCATTGCTGCC | | |  | AAGACATAAATGCCACCCTG | | |
| *Slc7a5* | AAGACACCCATGGAGTGTG | | |  | TCACAGAGAAGATAGCCTGC | | |
| *Downregulated* | |  |  |  |  |  |  |
| *Adora2a* | TTCCACTCCGGTACAATGG | | |  | CTGACTGCAGTTGTTCCAG | | |
| *Cadm1* | CAGGTGATGGACAGAATCTG | | |  | CCTTCAAAGGCCTGAAGTC | | |
| *Dagla* | CACCACCTGGTTTGTAATCC | | |  | TACAGCTCAGAAGGATGCC | | |
| *Fgfbp3* | TTCGCTCTACTCCAATAGGG | | |  | AAGGAGCATCCTAGGATCG | | |
| *Frk* | GAGAAGTTTGGGAAGGTCTG | | |  | CATTTGGATCCATTGAACCTG | | |
| *Gpd2* | GTTGAGCAGTATAGGATGGTG | | | | CGGAGCTGATAAATGAGGAG | | |
| *Gstm1* | CTGTTCTCTGCCTCAGGAG | | |  | ACATAGGTGACCTTGTCCC | | |
| *Nav2* | TGTCCAAAGAACAGATCGC | | |  | TCCTGATCTCTTCTGCAGAC | | |
| *Ptprt* | GGTTGTGGACATCTTCAACTG | | | | AACACGTACTGCTCCTCTG | | |
| *Tcf4* | CATGCAAGATGGCCATCAC | | |  | GAAGAATTGCCCAGCATCC | | |
|  |  |  |  |  |  |  |  |
| **For primary cortical neurons** | | |  |  |  |  |  |
| *Upregulated* | |  |  |  |  |  |  |
| *Anxa1* | CATCTTCGCAGAGTGTTTCAG | | | | ACCCTTCAGTTCCAGATCC | | |
| *Ar* | AAAGAAAGAATCCCACATCCTG | | | | CTCTTGCAATAGGCTGCAC | | |
| *Cd44* | GTACCTTACCCACCATGGAC | | |  | TTCCTTCTATGAACCCATACCTG | | |
| *Cgnl1* | ACCTGGAGTATGAGCTGGA | | |  | ACACCTTGTCCTCCATCTG | | |
| *Flt1* | TATTTGTTAGTGATGCAGGGAG | | | | GTCTTCCTTCCGTCATGTG | | |
| *Itga4* | CTGGAGGAGAGGGATAACC | | |  | CCCACAAGTCACGATAGAG | | |
| *Lama1* | CTACTATGGGAAGGTGACTGG | | | | GGGCTGAAACTGAAAGGAG | | |
| *Msn* | GAAGTTTGGTTCTTTGGTCTG | | | | TGCAGTCACCTTCTTATTGAG | | |
| *Plaur* | TTTCCATAGCAACCAGACCT | | |  | TGAAGATCCAGAACTGGGC | | |
| *Serpine1* | GAATACTCAGGATGCAGATGTC | | | | AAGTGAACCCTTTCCCAGAG | | |
| *Downregulated* | |  |  |  |  |  |  |
| *Dlx1* | AACTCAGTACTTAGCTCTGCC | | |  | AACCATATCTTGACCTGCGT | | |
| *Dlx2* | ACAATGTCTCCTACTCCGC | | |  | TCAAGGTCTTCCTTGTCCG | | |
| *Dlx6* | CAAACACAGGTGAAGATATGGT | | | | CTCGTGTGGGTTACTACCC | | |
| *Erbb4* | TGGACAACACTCTTCAGCA | | |  | GCCTTCAGCAGTACAATTCTC | | |
| *Gad1* | ACCTGTTTGCTCAAGATCTG | | |  | GTATGTCTACCACTTCCAGC | | |
| *Nxph1* | GTCTACTTGGTCACGTGTG | | |  | CTTTAGCGTGGATTTGCTG | | |
| *Rbms3* | CTTTGGACACGTGATATCCAC | | | | GACTCCATTCTGGCAAAGC | | |
| *Reln* | CTTTGGATTCGGGATCATGTC | | | | AGGTGACTCACATGAGAGG | | |
| *Slc6a1* | TGAACTCTTCATTGCTGCC | | |  | AAGACATAAATGCCACCCTG | | |

** Supplementary Fig. 1. Transcriptomic analysis reveals increased immune response and oligodendrocyte development in somatosensory cortex.**

**(A)** Immunoblotting of p-S6, c-S6, PTEN and GAPDH in the somatosensory cortices of *Pten*^+/-^ mice and littermate control (WT) of male and female at P30 and P42.

**(B)** Quantification of PTEN levels relative to GAPDH, and p-S6 levels relative to c-S6 as per cent of littermate control. Three-way ANOVA and Bonferroni *post hoc* multiple comparisons tests were used. F(1,8)=8.56, P<0.05 (for genotype) for p-S6/c-S6, F(1,8)=159.5, P<0.0001 (for genotype) for PTEN/GAPDH. N=3 per group. *, P<0.05; **, P< 0.01.

**(C)** Box plots of log normalized counts showed expression of top 10 most significant DEGs in All group.


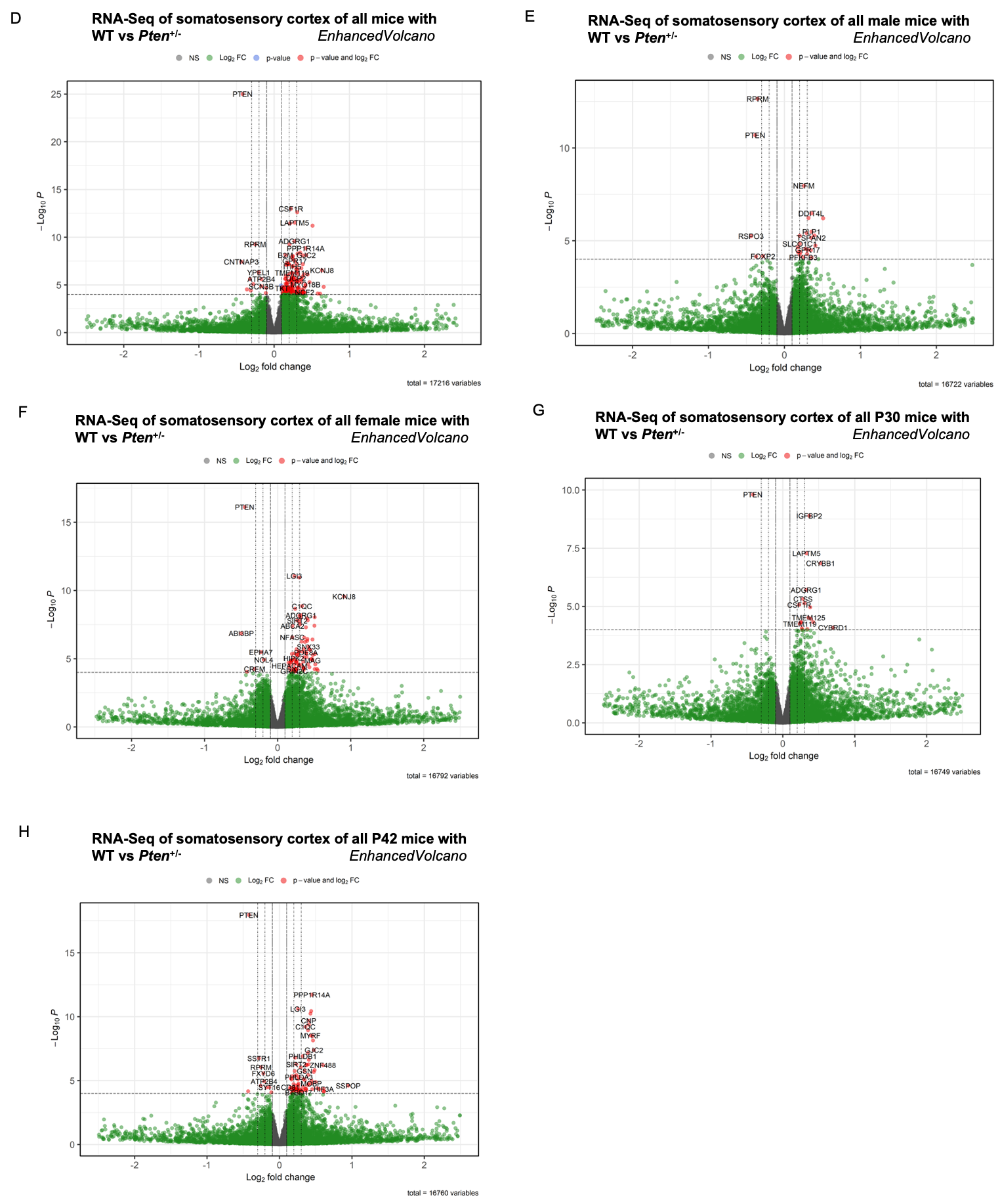


**Supplementary Fig. 1 cont’**

**(D-H)** Volcano plots of DEGs comparing somatosensory cortex of WT and *Pten*^+/-^ mice.

**
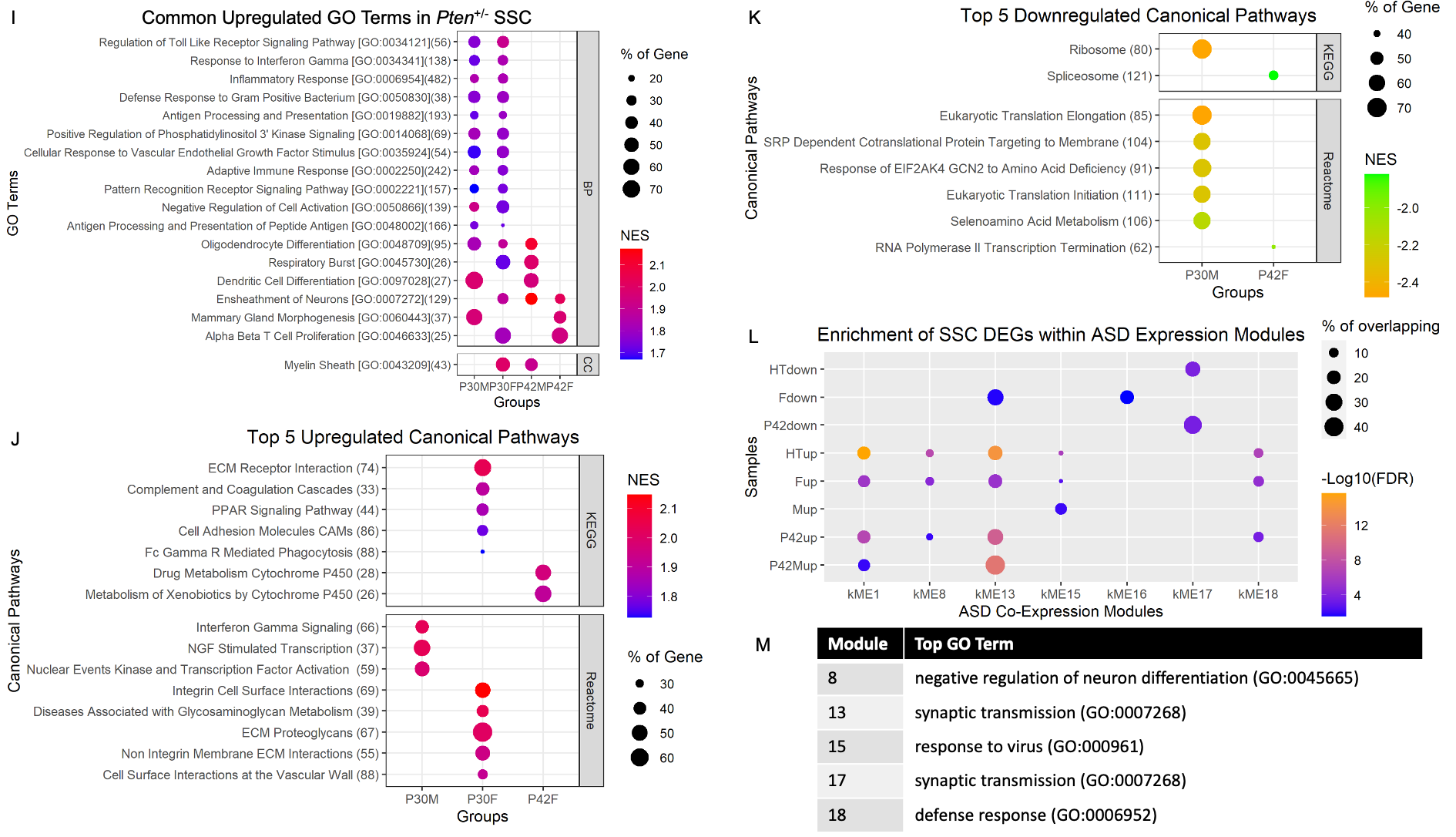
**

**Supplementary Fig. 1 cont’**

**(I)** Commonly upregulated GO terms shared amongst the groups.

**(J)** Top 5 upregulated canonical pathways in each group.

**(K)** Top 5 downregulated canonical pathways in each group.

**(L)** Enrichment of upregulated and downregulated DEGs in each group within ASD co-expression modules. Only modules enriched (FDR<0.05) with at least one group of DEGs were displayed here. Statistical analysis of enrichment was calculated by FDR-corrected cumulative hypergeometric test. HTdown: downregulated DEGs in *Pten* heterozygous (HT) mice amongst all groups; HTup: upregulated DEGs in HT mice amongst all groups. **(M)** Table shows the top GO term of each enriched module.


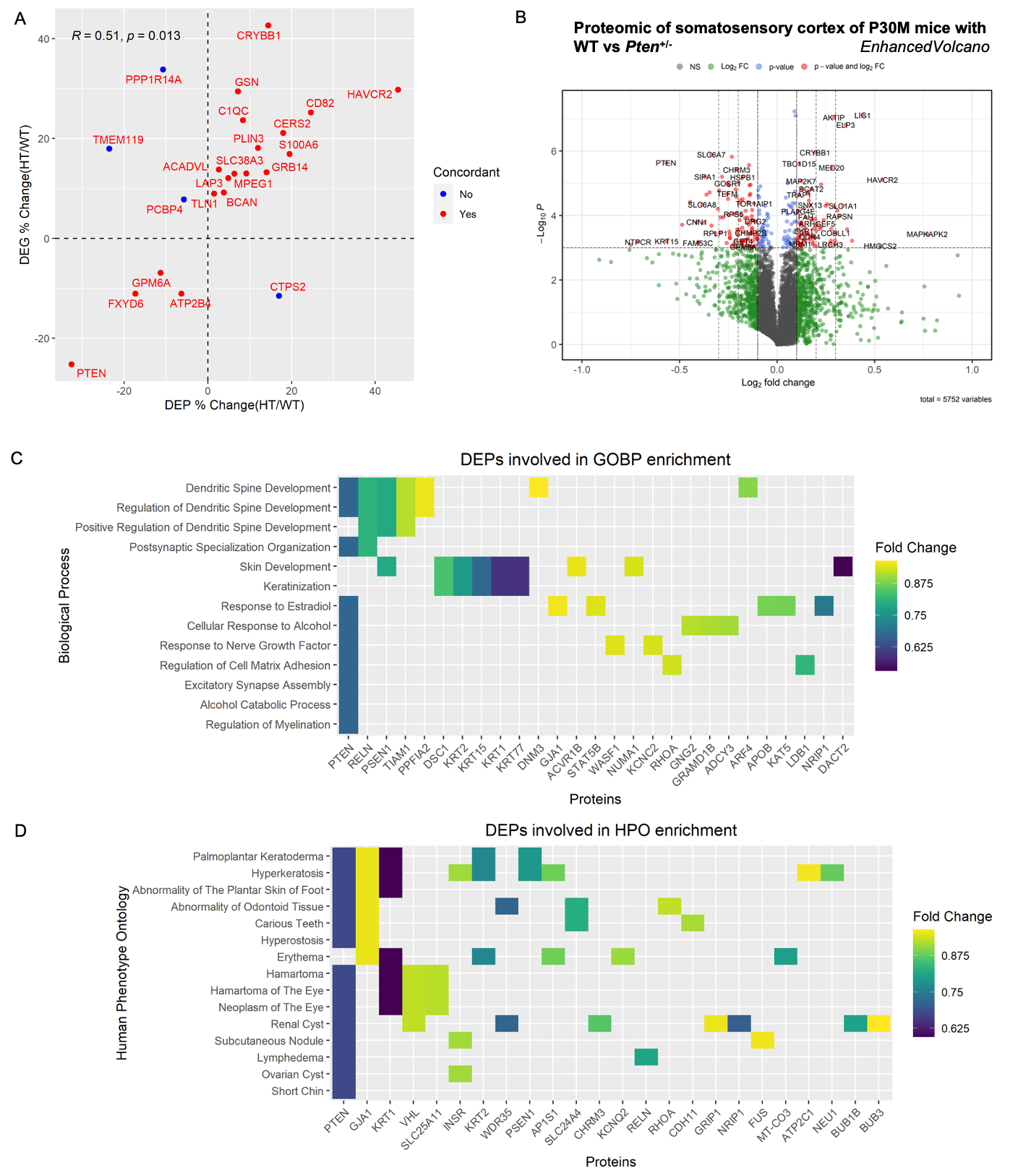


**Supplementary Fig. 2. Proteomic analysis of somatosensory cortex reveals perturbation of dendritic spine development, keratinization and hamartoma signatures.**

**(A)** Percentage change dimensionality comparison of DEGs and DEPs (HT/WT). R: Spearman’s correlation coefficient.

**(B)** Data are shown as Volcano plot.

Heatmaps showed DEPs with FDR < 0.05 shared amongst enriched

**(C)** GOBP terms and,

**(D)** HPO terms.

**Supplementary Fig. 3. Transcriptomic analysis of *Pten^+/-^* haploinsufficient primary neural cells.** Immunoblotting of PTEN, p-AKT(S473), c-AKT, p-S6, c-S6, p-ERK, c-ERK, NESTIN, NF68 and β-actin in the primary cultures of

**(A)** NPC,

**(C)** astrocyte and

**(E)** neuron of *Pten*^+/-^ mice and wild-type littermate control (WT) at E12.5, P2 and E16.5, respectively. Hippocampal lysate from wild-type mouse at P19 (P19 HC) was used as control for the corresponding presence of proteins at correct size. Quantification of PTEN relative to β-actin and selected signaling molecules of PI3K pathway in

**(B)** NPC,

**(D)** astrocyte and

**(F)** neuron. Unpaired t-test was used for statistical analysis. ** indicates P-values < 0.01 and **** < 0.0001.


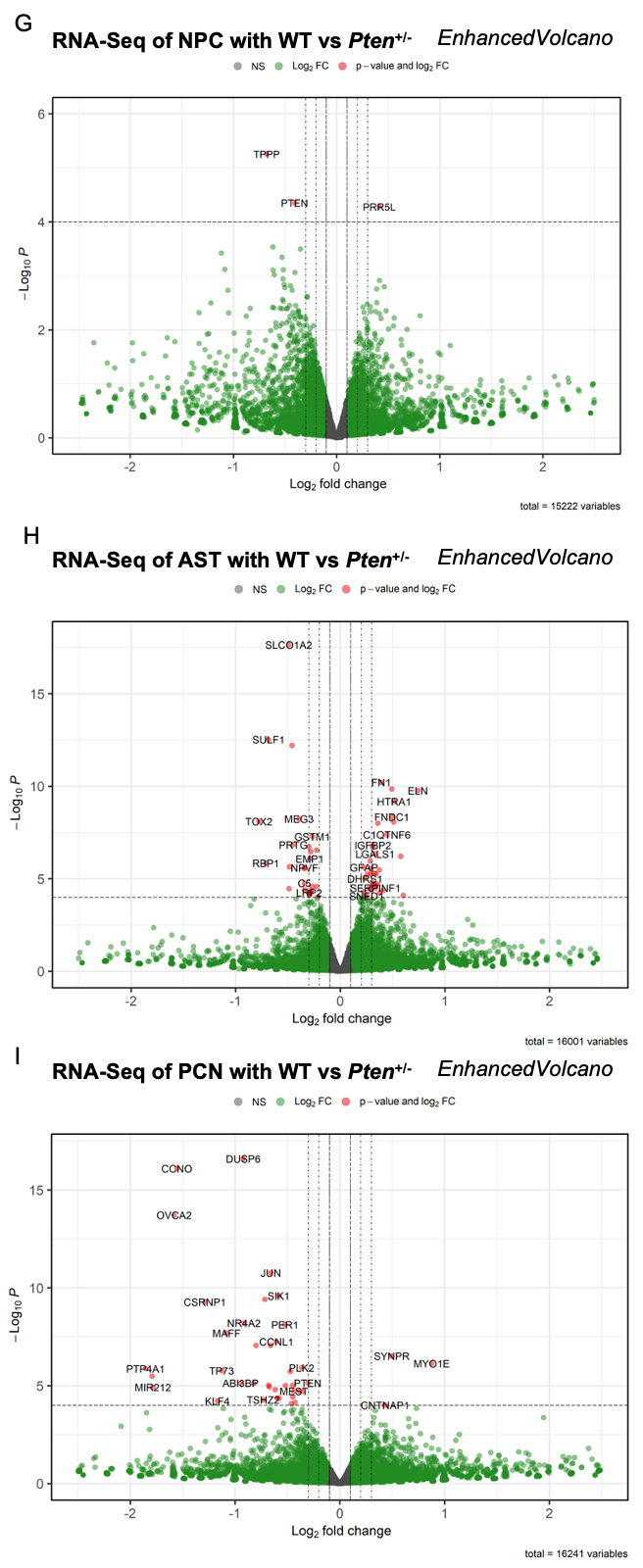


**Supplementary Fig. 3 cont’** Volcano plots of DEGs between WT and *Pten*^+/-^ from

**(G)** neural progenitor cells (NPC).

**(H)** primary astrocytes (AST).

**(I)** primary cortical neurons (PCN).

** Supplementary Fig. 4. Side-view of brains of *Pten*-deficient mice at different developmental stages.**

Representative side view of brain with skull removed at E14, E16.5 and P0 of *Ctrl*, *Nes-HET*, and *Nes-KO* littermate mice. Scale bar = 1mm.

**Supplementary Fig. 5. Transcriptomic analysis of *Pten* knockout primary neural cells uncover neural cell-specific signatures.**

**(A)** Top 10 most upregulated canonical pathways (FDR<0.05) enriched in *Pten* knockout primary neural cells.

**(B)** Top 10 most downregulated canonical pathways (FDR<0.05) enriched in *Pten* knockout primary neural cells.

**Supplementary Fig. 5 cont’** Volcano plots of DEGs between *Ctrl* and *Nes-KO* from

**(C)** neural progenitor cells (NPC),

**(D)** primary astrocytes (AST), and

**(E)** primary cortical neurons (PCN).

**Supplementary Fig. 6. Immunblotting of inhibitory neurons related protein in *Pten* knockout primary neuron (PCN).**

Immunoblotting of PTEN, RELN, ERBB4, SST, NPY, and β-actin of primary cultures of neuron at DIV5 and DIV14 from *Pten* knockout (*Nes-KO*)(n=5/n=6) and wild-type littermate control (*Ctrl*) (n=3/n=3) derived from embryonic mice at E16.5.

**Supplementary table list**

**ST1**.SSCvsASDmodules.xlsx – Corresponds to supplementary figure 1L. It is the genelists of DEGs from the somatosensory cortex of mice with WT and heterozygous Pten enriched within ASD co-expression modules.

**ST2a**.SSC.DEP&DEG.UP.xlsx – Corresponds to figure 2D. It is the genelists of upregulated DEGs from the somatosensory cortex of mice with WT and heterozygous Pten overlapped with upregulated DEP.

**ST2b**.SSC.DEP&DEG.down.xlsx – Corresponds to figure 2E. It is the genelists of downregulated DEGs from the somatosensory cortex of mice with WT and heterozygous Pten overlapped downregulated DEP.

**ST2c**.DEPvsGOBP.genelists.xlsx – Corresponds to supplementary figure 2C. It is the GSEA analysis of DEP list enriched with GO (biological properties) terms, FDR < 0.05.

**ST2d**.DEPvsHPO.genelists.xlsx – Corresponds to supplementary figure 2D. It is the GSEA analysis of DEP list enriched with HPO, FDR < 0.05.

**ST3a**.DEPvsNeuralCells.up.xlsx – Corresponds to figure 6C. It is the genelists of upregulated DEGs from different neural cells with Pten loss overlapped with upregulated DEP.

**ST3b**.DEPvsNeuralCells.down.xlsx – Corresponds to figure 6D. It is the genelists of downregulated DEGs from primary different neural cells with Pten loss overlapped with downregulated DEP.

**ST3c**.DEP_filtered_DEGs.genelists.xlsx – Corresponds to figure 6E. It is the genelists curated from DEP, DEGs from somatosensory cortex of mice and DEGs from different primary neural cells with Pten loss. These lists are used for enrichment analysis with GWAS genelists of neurological diseases and traits.

**ST3d**.GWAS genelists.csv – Corresponds to figure 6E. It is the GWAS genelists of neurological diseases and traits.

**ST3e**.DEP.filteredDEGs.vs.GWAS.genelists.xlsx – Corresponds to figure 6E. It is the genelists from ST3c.DEP_filtered_DEGs.genelists overlapped with GWAS genelists of neurological diseases and traits.

**ST4**.xlsx – Summary of models, genotypes, experiments and main findings used in each figure.

**Reference**

1. Currle DS, Hu JS, Kolski-Andreaco A, Monuki ES. Culture of mouse neural stem cell precursors. J Vis Exp. 2007(2):152.

2. Nunez Rodriguez N, Lee IN, Banno A, Qiao HF, Qiao RF, Yao Z, et al. Characterization of R-ras3/m-ras null mice reveals a potential role in trophic factor signaling. Mol Cell Biol. 2006;26(19):7145-54.

3. Hilgenberg LG, Smith MA. Preparation of dissociated mouse cortical neuron cultures. J Vis Exp. 2007(10):562.

4. Kim D, Paggi JM, Park C, Bennett C, Salzberg SL. Graph-based genome alignment and genotyping with HISAT2 and HISAT-genotype. Nat Biotechnol. 2019;37(8):907-15.

5. Liao Y, Smyth GK, Shi W. featureCounts: an efficient general purpose program for assigning sequence reads to genomic features. Bioinformatics. 2014;30(7):923-30.

6. Love MI, Huber W, Anders S. Moderated estimation of fold change and dispersion for RNA-seq data with DESeq2. Genome Biol. 2014;15(12):550.

7. Durinck S, Spellman PT, Birney E, Huber W. Mapping identifiers for the integration of genomic datasets with the R/Bioconductor package biomaRt. Nat Protoc. 2009;4(8):1184-91.

8. Mootha VK, Lindgren CM, Eriksson KF, Subramanian A, Sihag S, Lehar J, et al. PGC-1alpha-responsive genes involved in oxidative phosphorylation are coordinately downregulated in human diabetes. Nat Genet. 2003;34(3):267-73.

9. Subramanian A, Tamayo P, Mootha VK, Mukherjee S, Ebert BL, Gillette MA, et al. Gene set enrichment analysis: a knowledge-based approach for interpreting genome-wide expression profiles. Proc Natl Acad Sci U S A. 2005;102(43):15545-50.

10. Liberzon A, Subramanian A, Pinchback R, Thorvaldsdottir H, Tamayo P, Mesirov JP. Molecular signatures database (MSigDB) 3.0. Bioinformatics. 2011;27(12):1739-40.

11. Wang M, Zhao Y, Zhang B. Efficient Test and Visualization of Multi-Set Intersections. Sci Rep. 2015;5:16923.

12. Supek F, Bosnjak M, Skunca N, Smuc T. REVIGO summarizes and visualizes long lists of gene ontology terms. PLoS One. 2011;6(7):e21800.

13. Wen B, Zhou R, Feng Q, Wang Q, Wang J, Liu S. IQuant: an automated pipeline for quantitative proteomics based upon isobaric tags. Proteomics. 2014;14(20):2280-5.

**Supplementary full figures –** due to the scarcity of protein samples from pairing littermates, full membranes were closely cropped in order to fully utilize a membrane to probe for multiple proteins of different sizes.

**Figure 4. Aberrant PI3-K signalling in *Pten*-knockout neural cells.** Immunoblotting of the indicated proteins in **(B)** NPCs, **(E)** ASTs and **(H)** neurons of *Pten*-knockout (*Nes-KO*) and littermate control (*Ctrl*) mice at E12.5, P0 and E16.5, respectively.

**Figure 4B**

PTEN PTEN (Overexposed)


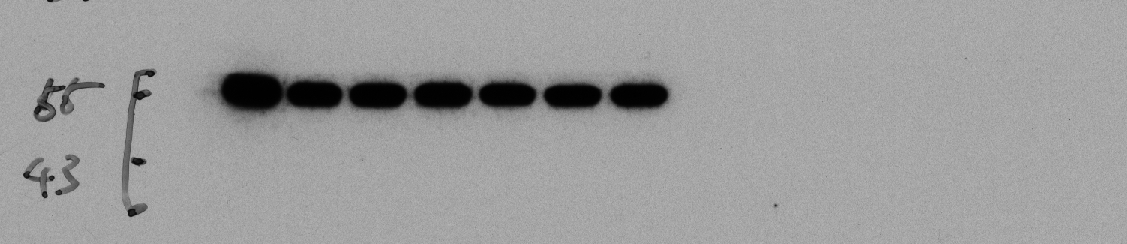


p-AKT p-AKT (Overexposed)


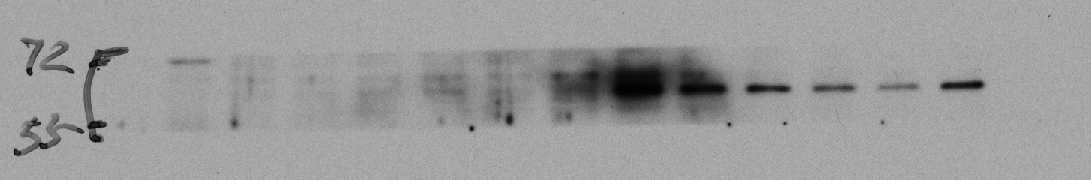


c-AKT c-AKT (Overexposed)


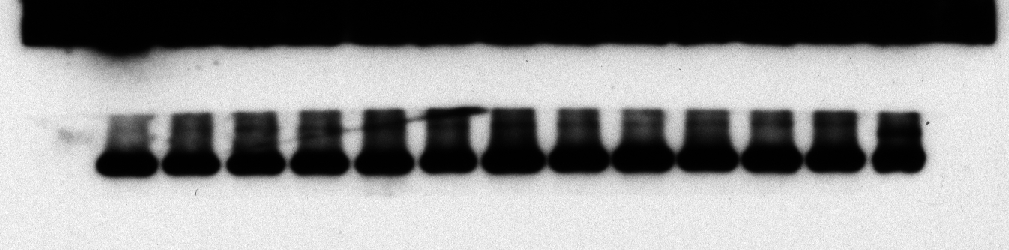


p-S6 p-S6 (Overexposed)


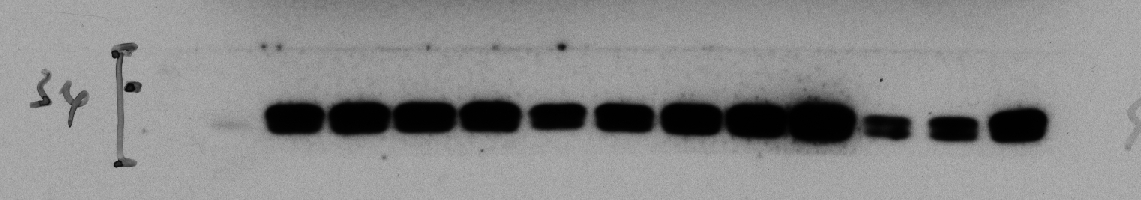


c-S6 c-S6 (Overexposed)


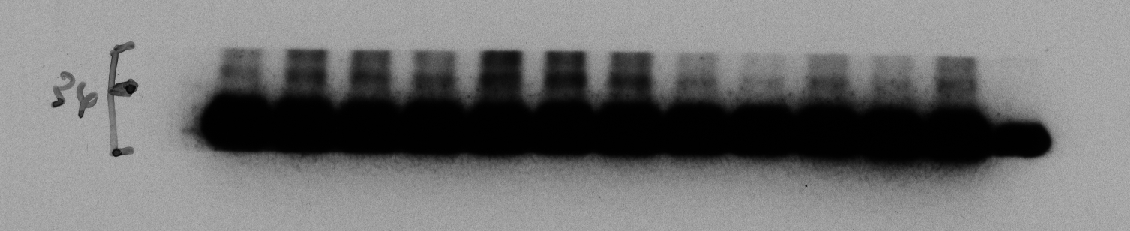


p-GSK3-β p-GSK3-β (Overexposed)


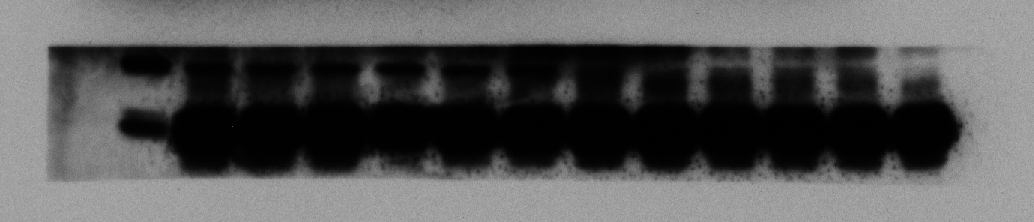


c-GSK3-β c-GSK-3β (Overexposed)


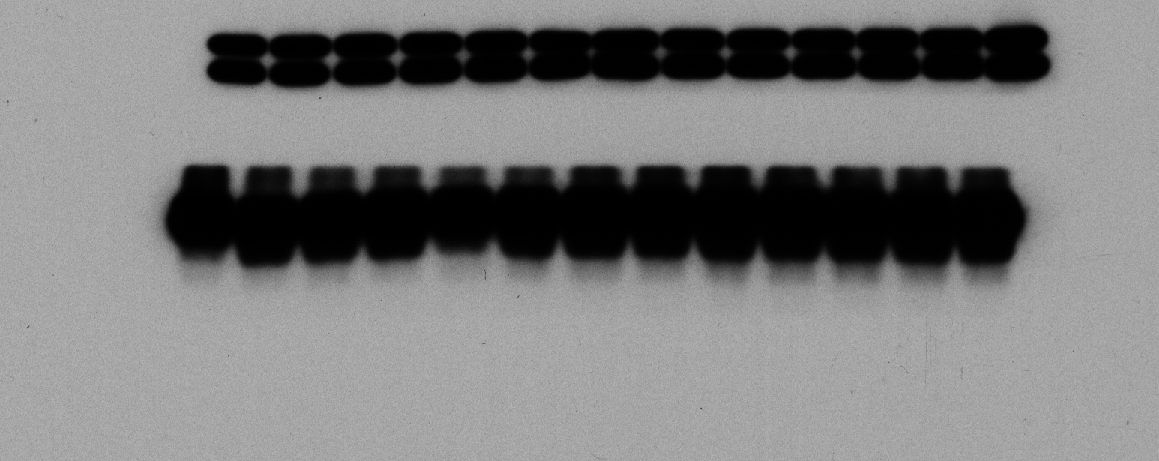


p-mTOR p-mTOR (Overexposed)


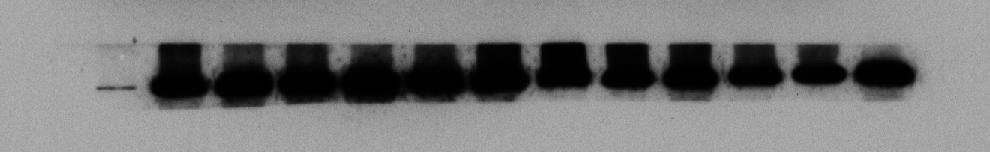


c-mTOR c-mTOR (Overexposed)


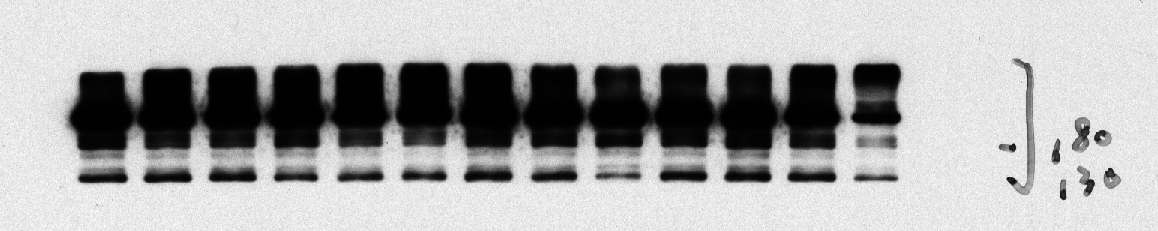


p-FOXO1/ (Overexposed)

c-FOXO c-FOXO (Overexposed)


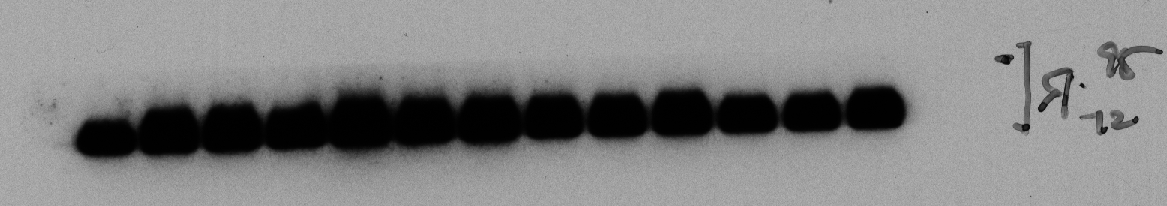


p-ERK p-ERK (Overexposed)


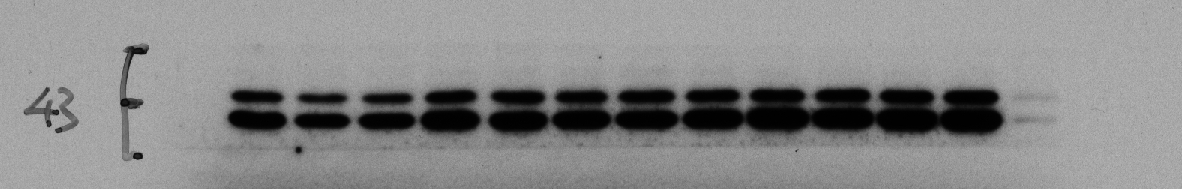


c-ERK c-ERK (Overexposed)


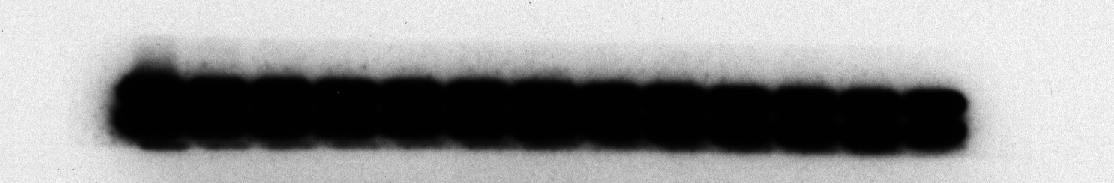


NESTIN NESTIN (Overexpressed)


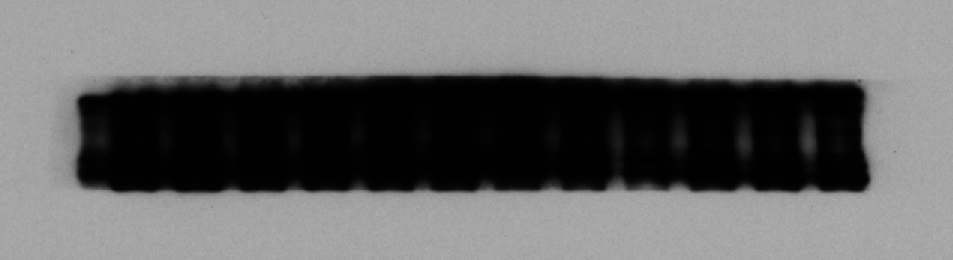


NeuN NeuN (Overexposed)


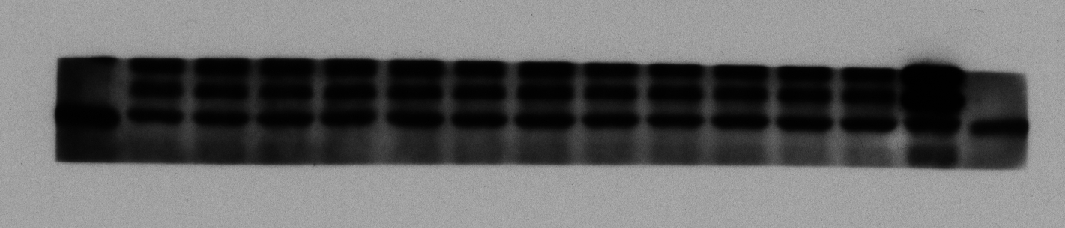


GFAP (this blot was first probed with anti-c-ERK antibody (the lower two bands) and then reprobed with anti-GFAP antibody. The 50 KDa GFAP band is indicated by arrow.

GAPDH/ (Overexposed)

**Fig. 4E**

PTEN, p-S6 (Overexposed)

p-AKT / (Overexposed)

c-AKT, c-S6 c-AKT, c-S6 (Overexposed)


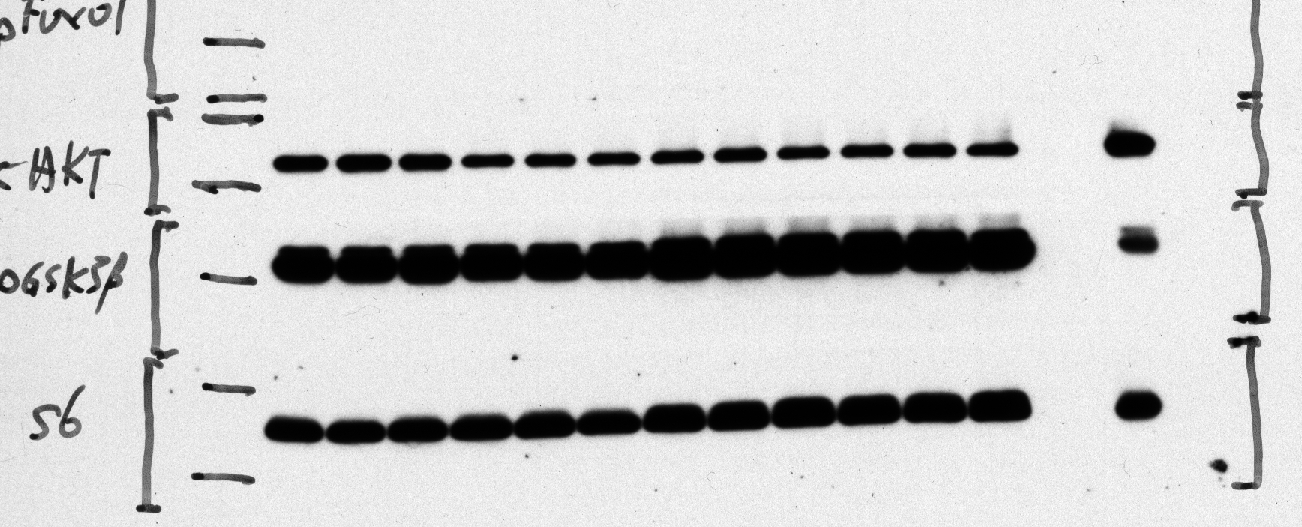


p-GSK-3β p-GSK-3β (Overexposed)


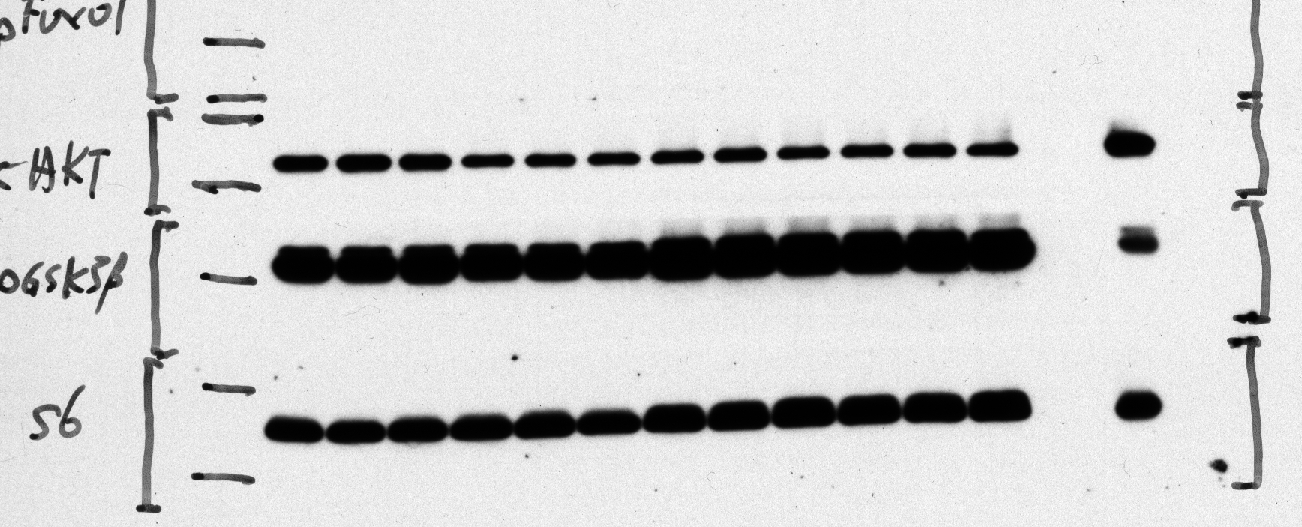


c-GSK3-β, c-mTOR c-GSK3-β, c-mTOR (Overexposed)


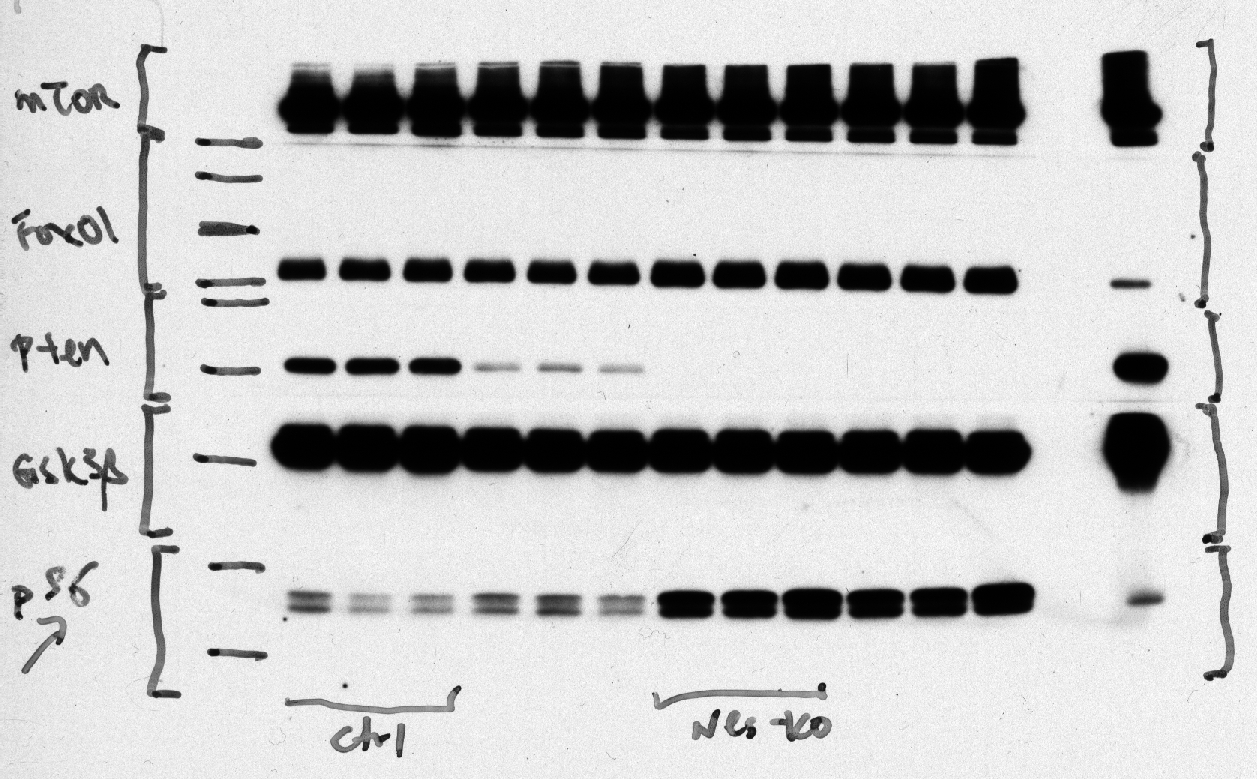


p-mTOR p-mTOR (Overexposed)


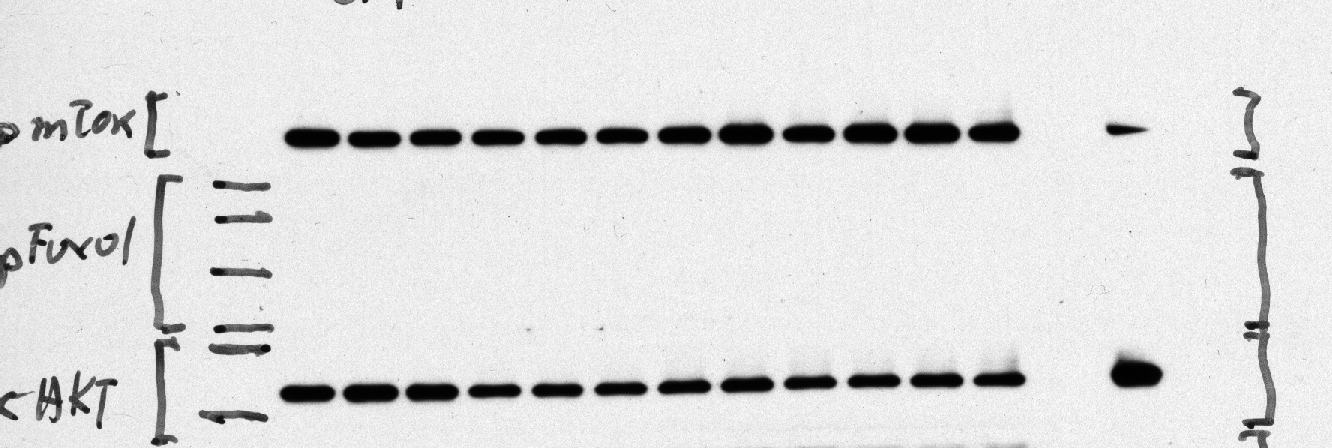


p-FOXO1 p-FOXO1 (Overexposed)


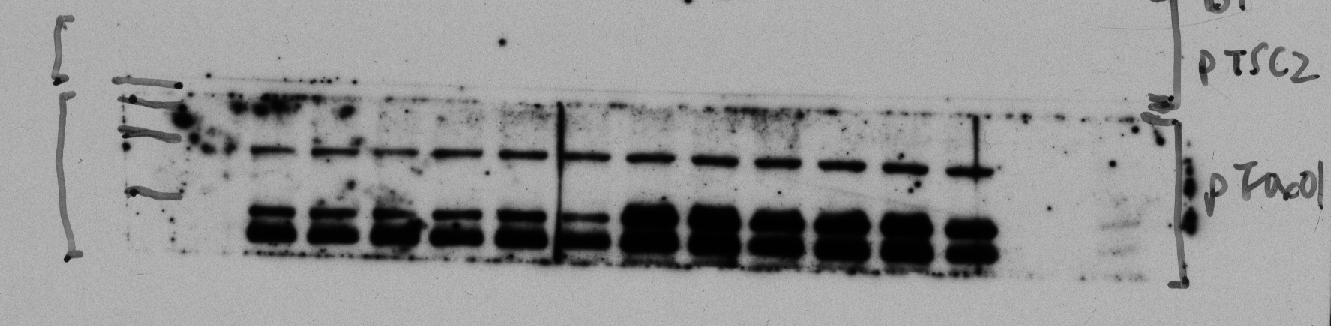


c-FOXO1 c-FOXO1 (Overexposed)


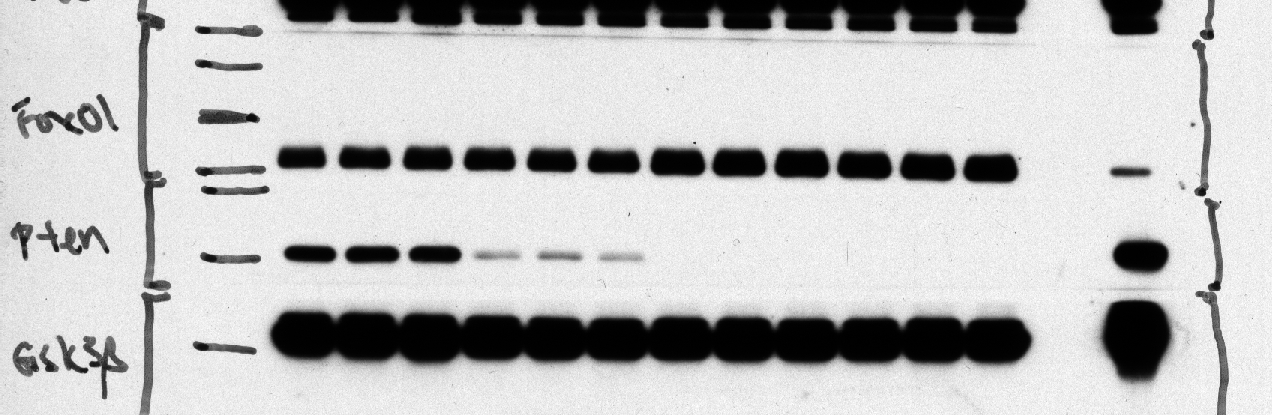


p-ERK p-ERK (Overexposed)


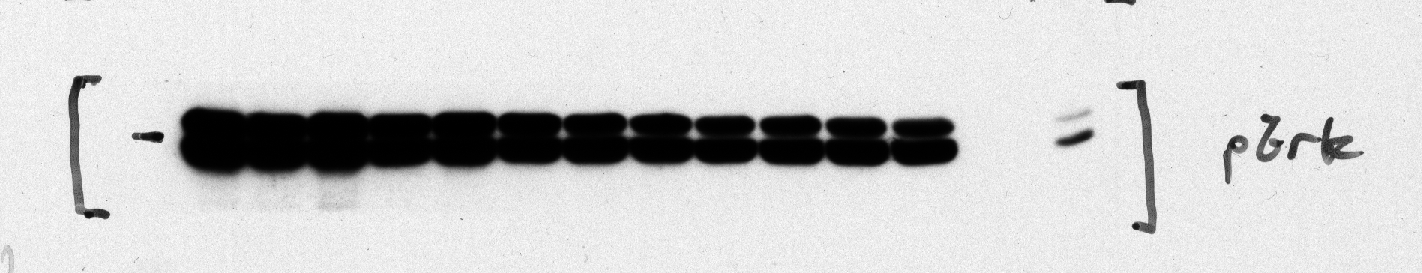


c-ERK c-ERK (Overexposed)


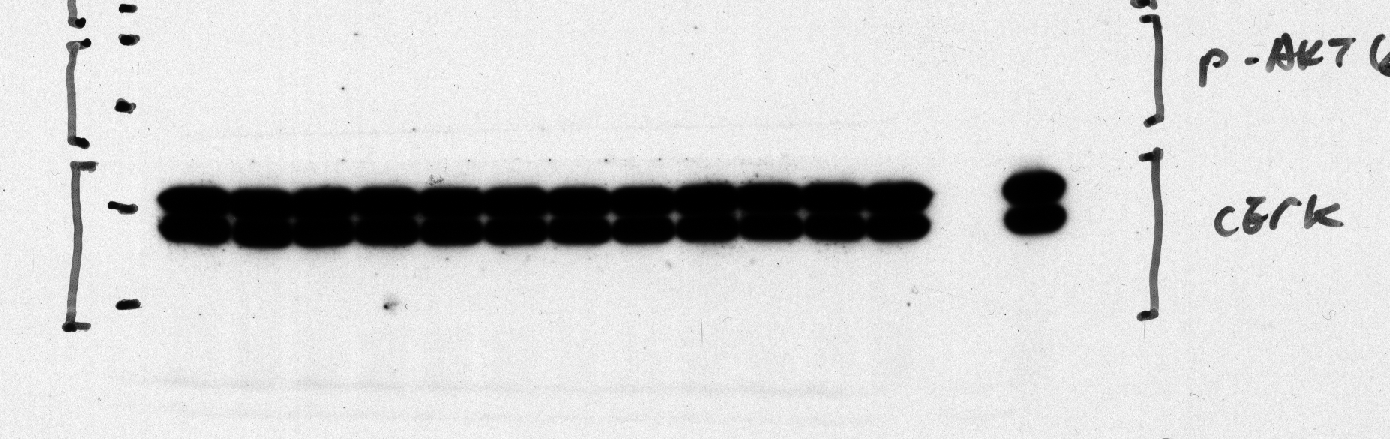


GFAP GFAP (Long exposure)


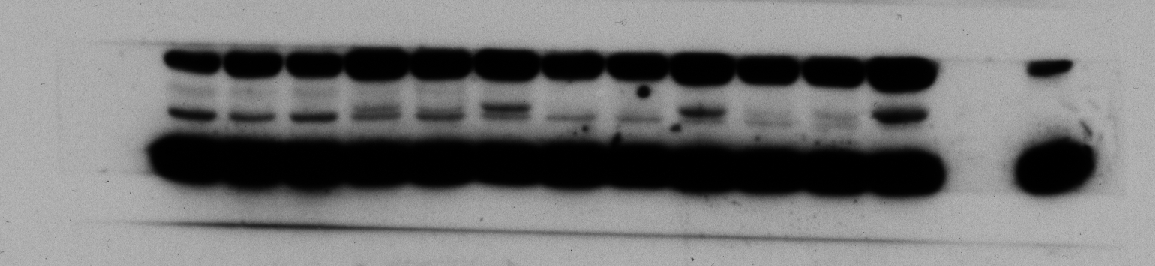


GAPDH GAPDH (Overexposed)


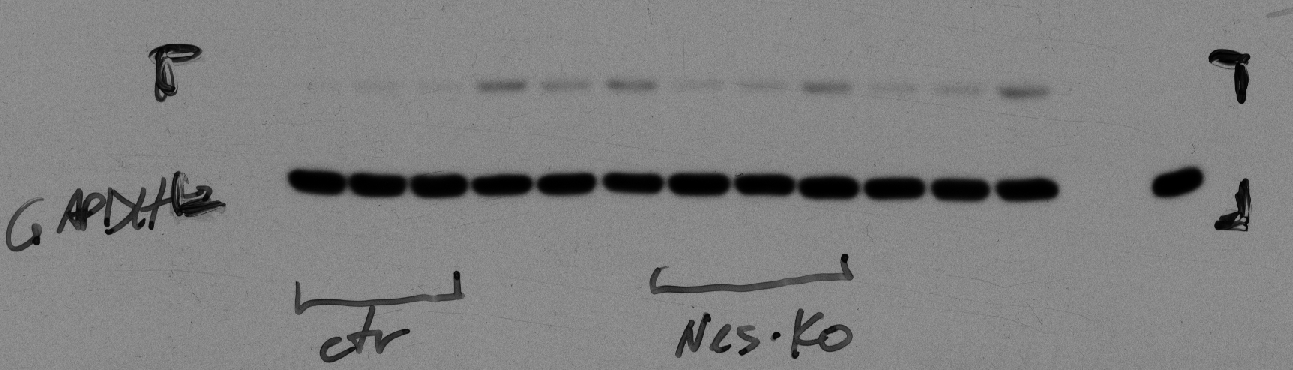


**Figure 4H**

PTEN (Margin marked)

p-AKT / (Overexposed)

c-AKT c-AKT (Overexposed)


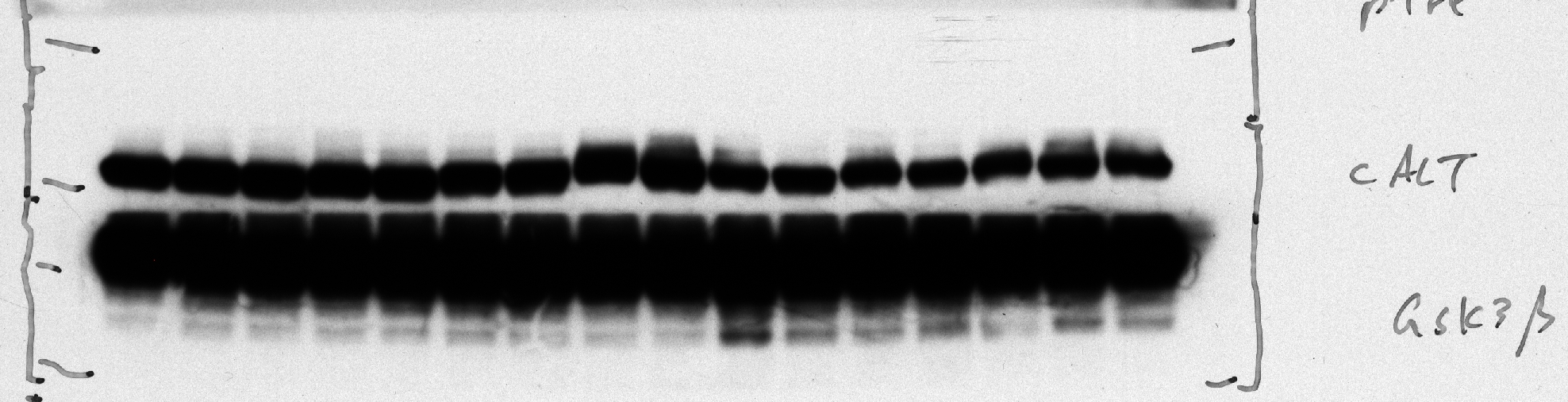


p-S6 p-S6 (Overexposed)


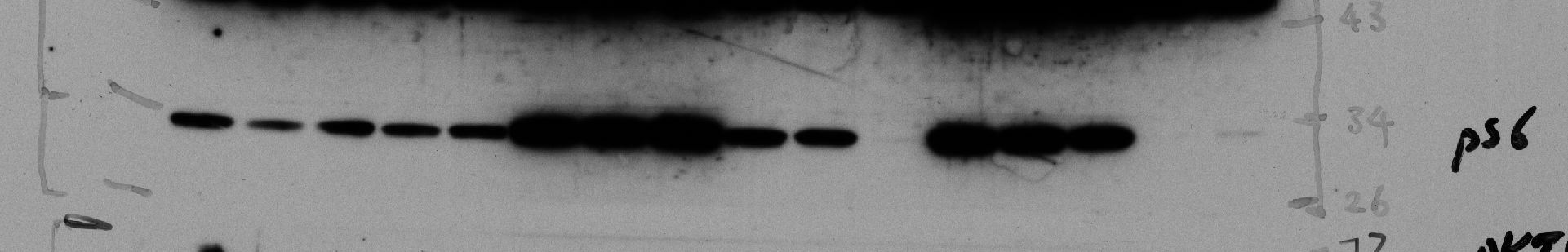


c-S6 c-S6 (Overexposed)


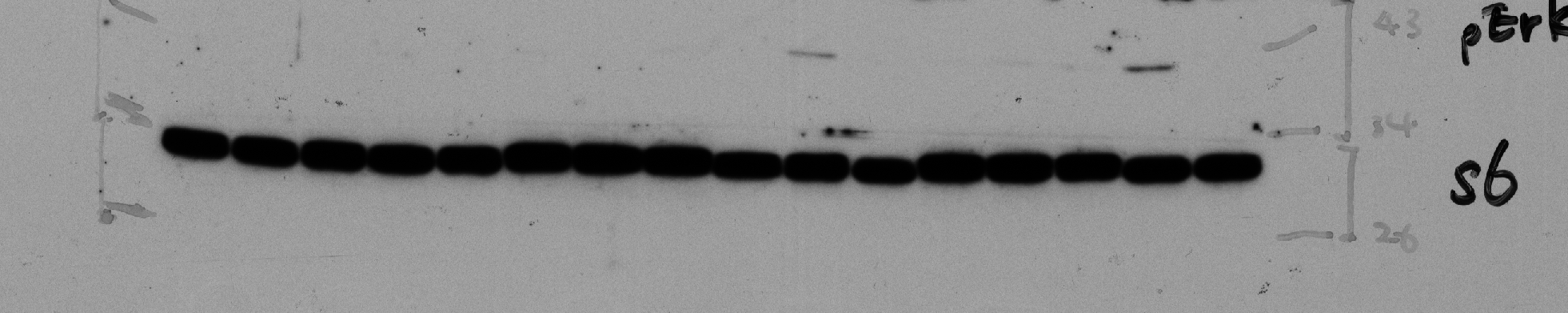


p-GSK-3β p-GSK-3β (Overexposed)


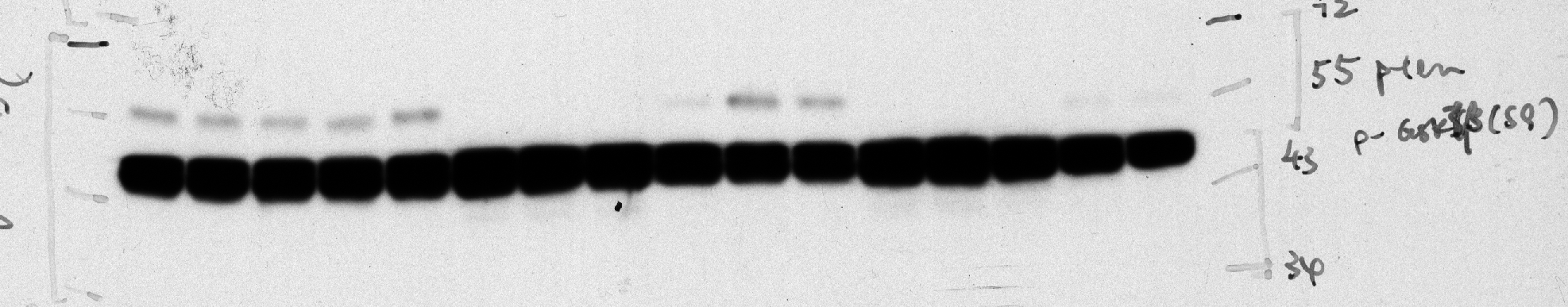


c-GSK-3β (Margin marked)

p-ERK (Margin marked)

c-ERK c-ERK (Overexposed)


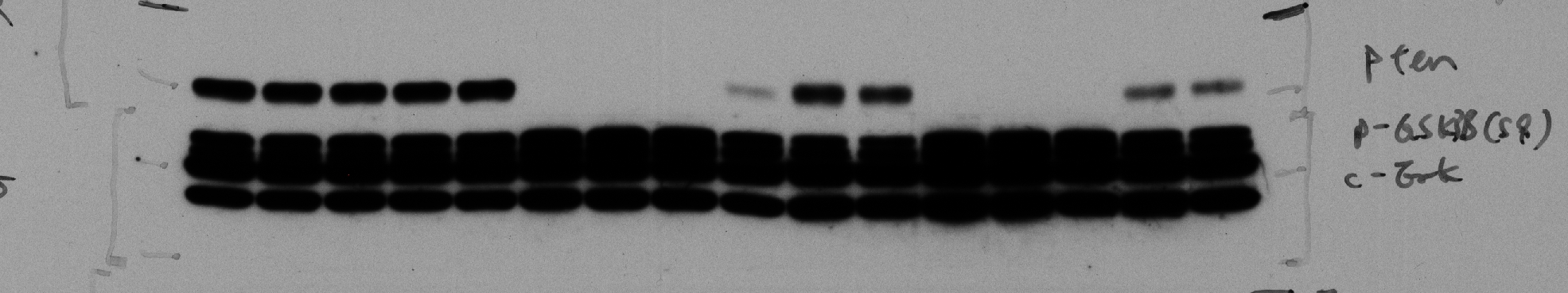


NF68 NF68 (Overexposed)


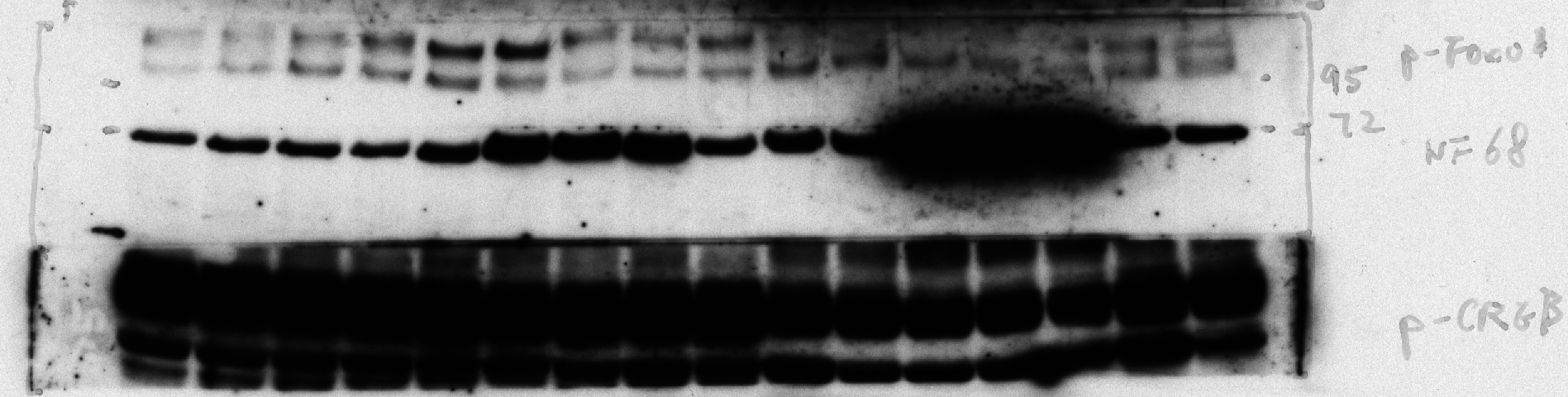


Actin Actin (margin marked)


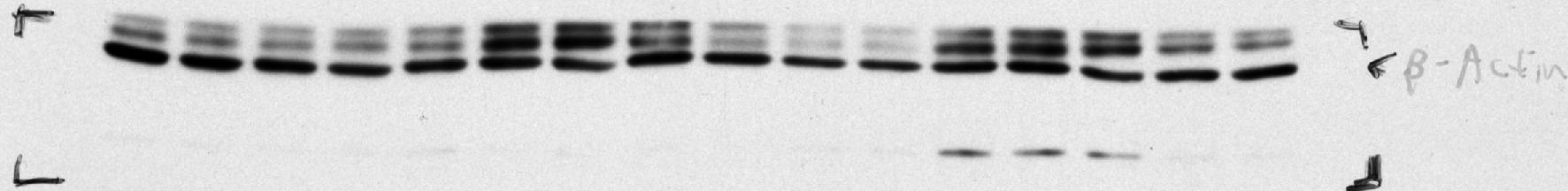


**Supplementary Fig. 1. Transcriptomic analysis reveals increased immune response and oligodendrocyte development in somatosensory cortex.**

**(A)** Immunoblotting of p-S6, c-S6, PTEN and GAPDH in the somatosensory cortices of *Pten*^+/-^ mice and littermate control (WT) of male and female at P30 and P42.

PTEN (P30) PTEN (P30) (Overexposed)


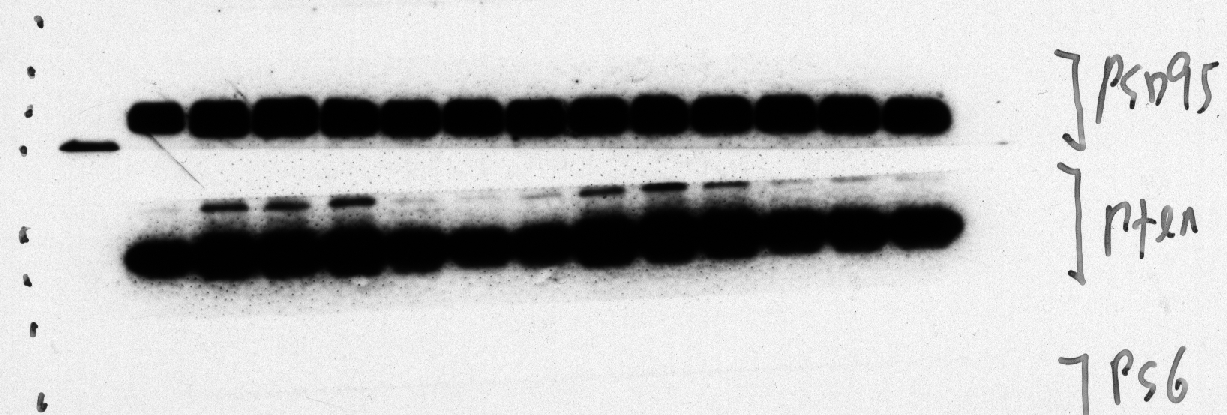


PTEN (P42) PTEN (P42) (Overexposed)


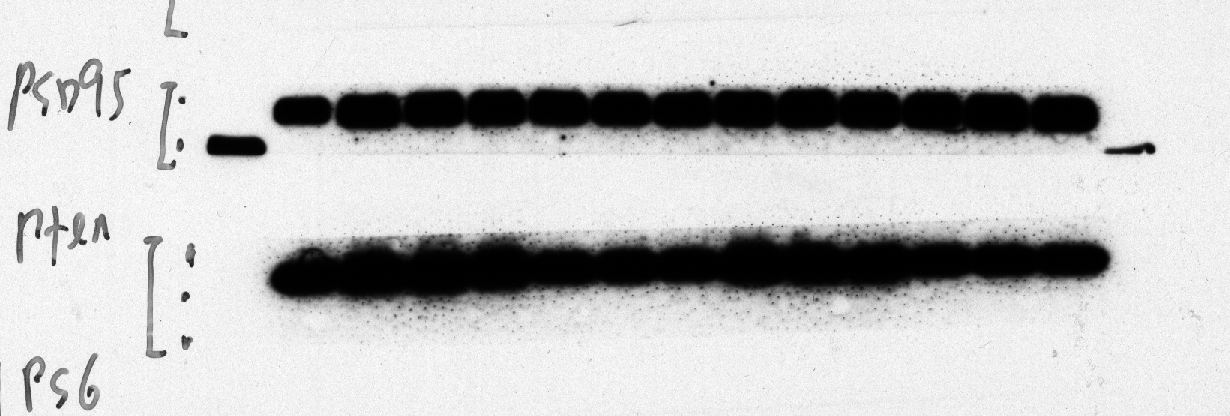


GAPDH (P30) & GAPDH (P42)

p-S6 (P30) p-S6 (P30 (margin marked)


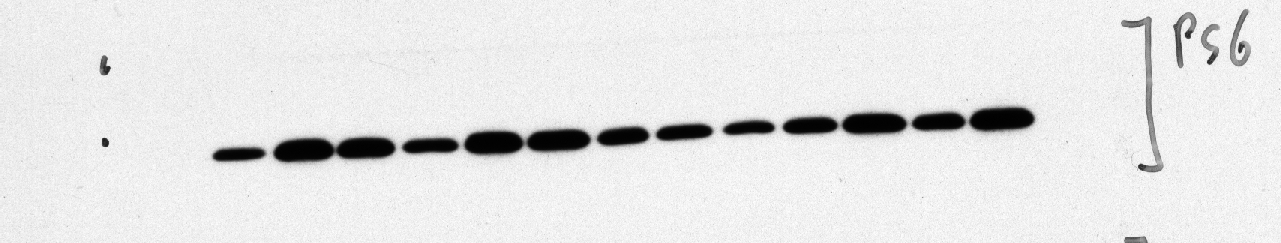


c-S6 (P30) c-S6 (P30) (margin marked)


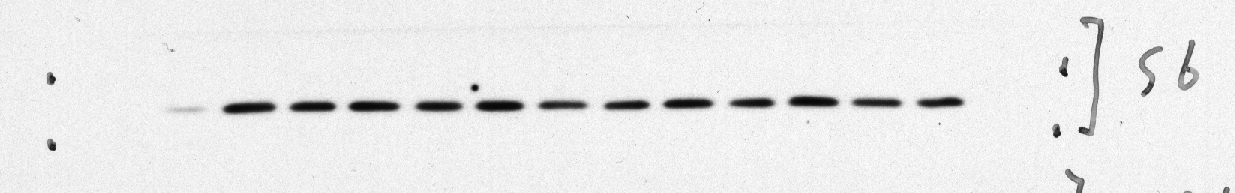


p-S6 (P42) p-S6 (P42) (Margin marked)


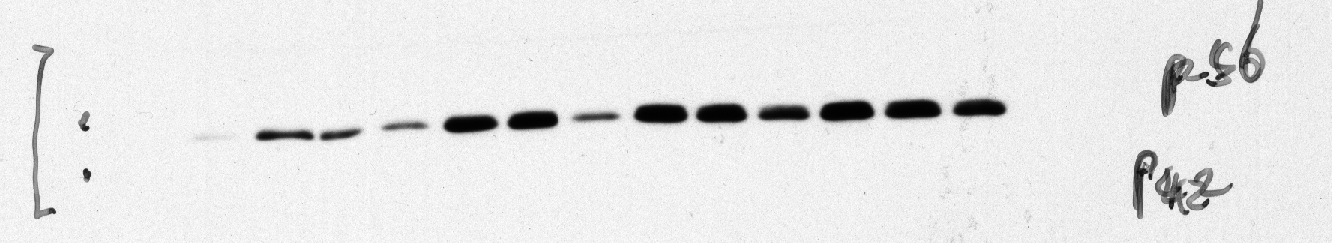


c-S6 (P42) (Overexposed)

**Supplementary Fig. 3. Transcriptomic analysis of *Pten^+/-^* haploinsufficient primary neural cells.** Immunoblotting of PTEN, p-AKT(S473), c-AKT, p-S6, c-S6, p-ERK, c-ERK, NESTIN, NF68 and β-actin in the primary cultures of

**(A)** NPC, **(C)** astrocyte, and **(E)** neuron

**Fig. S3A**

PTEN PTEN (Overexposed)


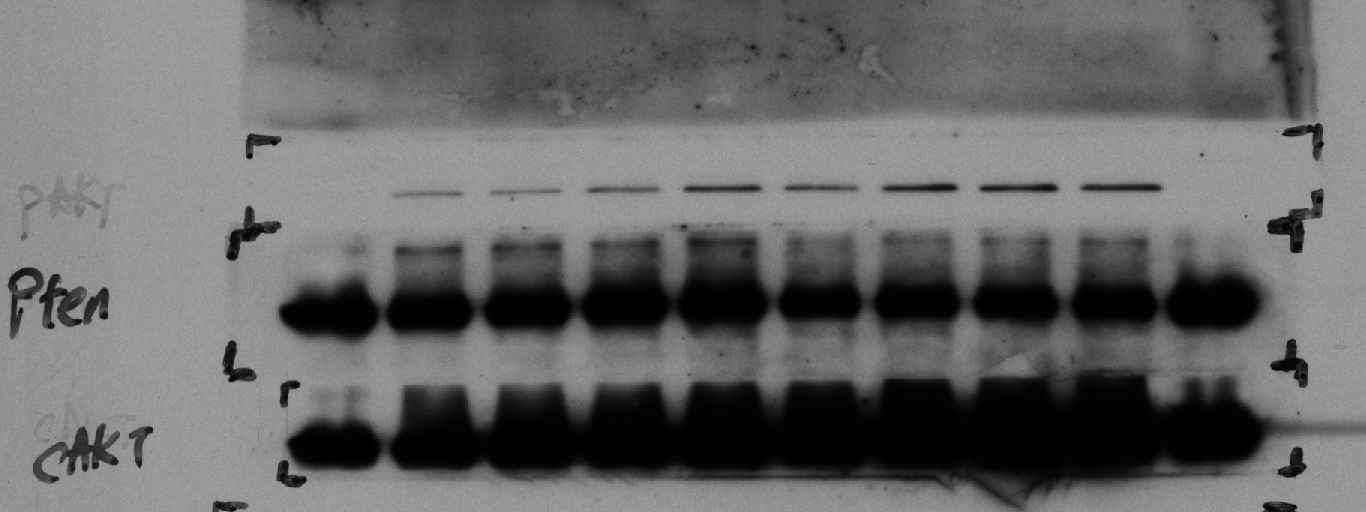


p-AKT (Overexposed)

c-AKT c-AKT (Overexposed)


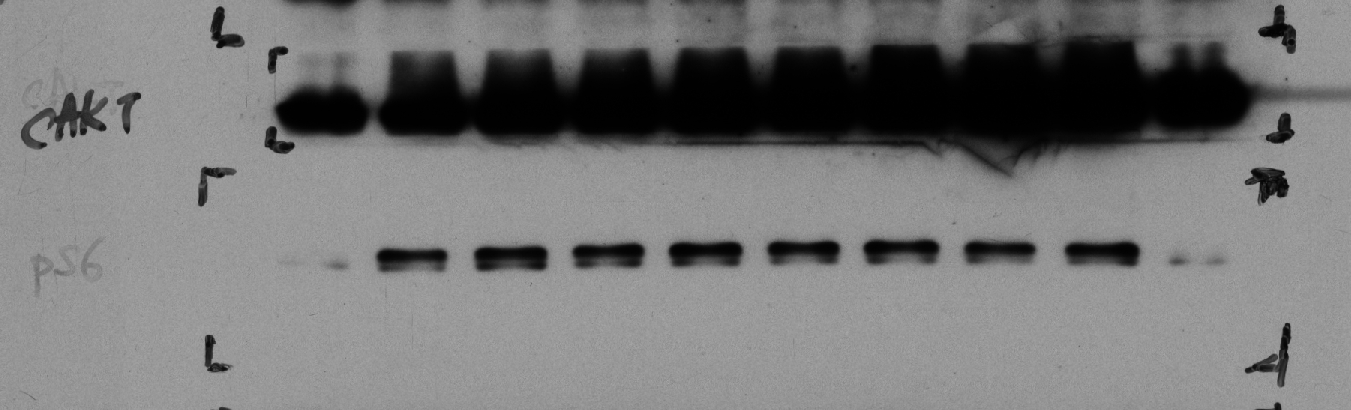


p-S6 p-S6 (Overexposed)


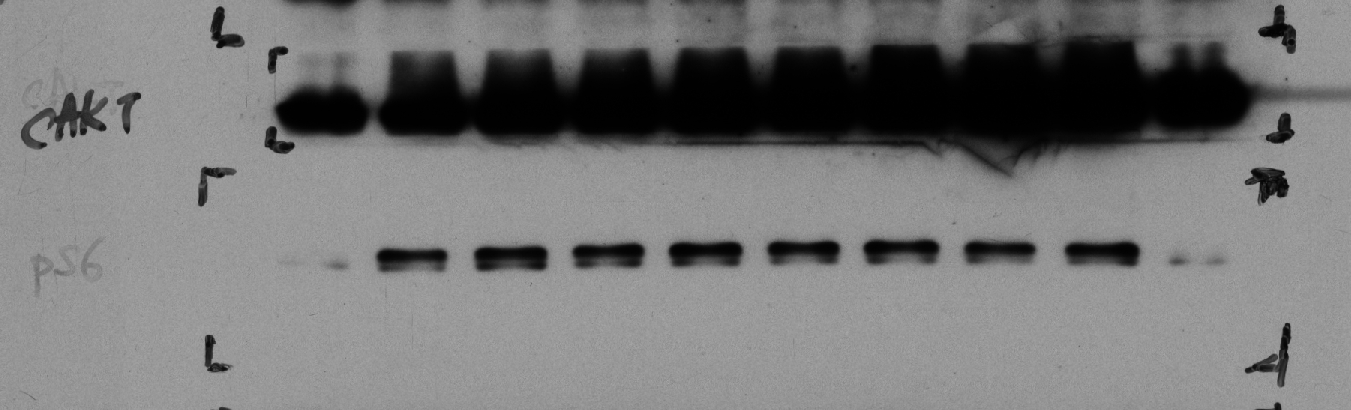


c-S6/p-ERK c-S6/p-ERK (overexposed)


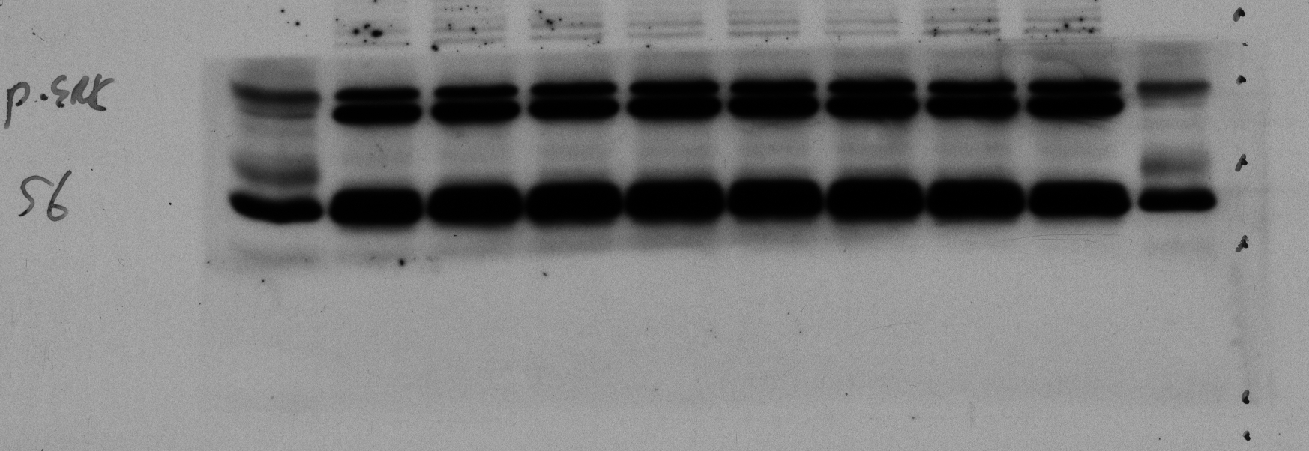


c-ERK c-ERK (Overexposed)


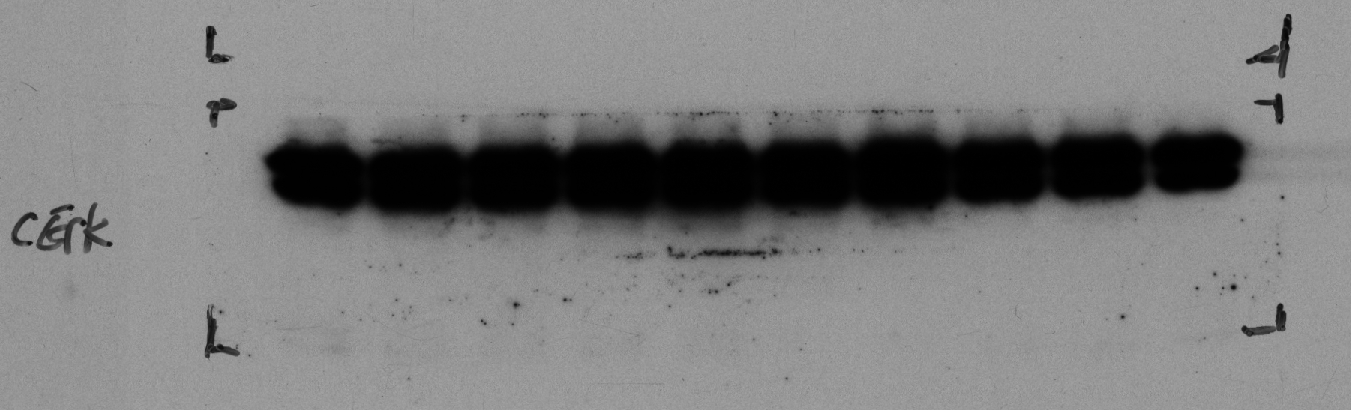


NESTIN NESTIN (Overexposed)


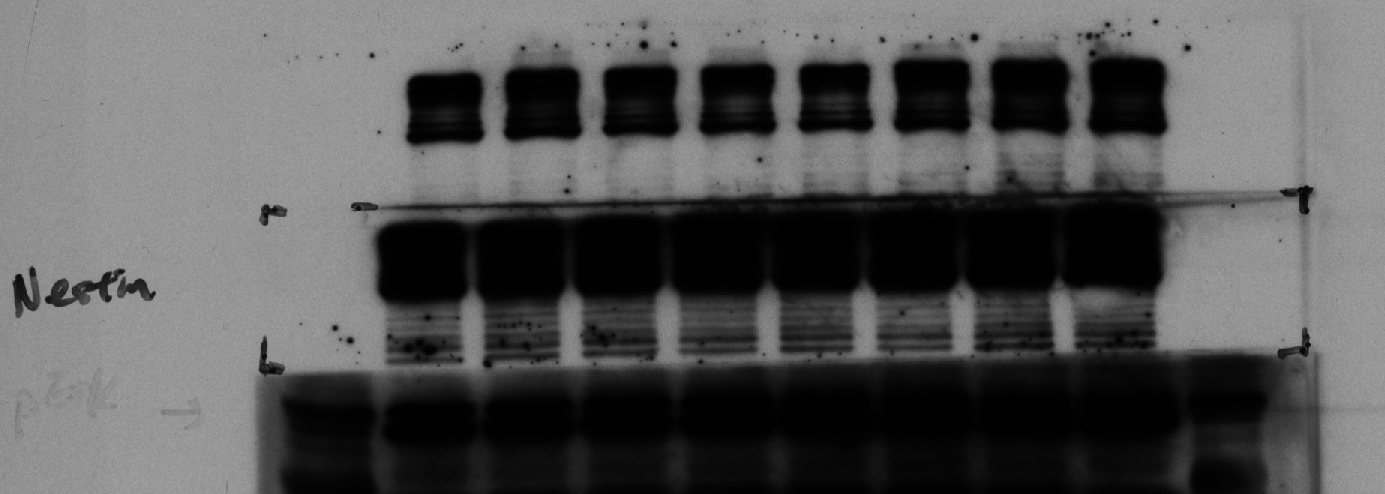


NF-68 NF-68 (Overexposed)


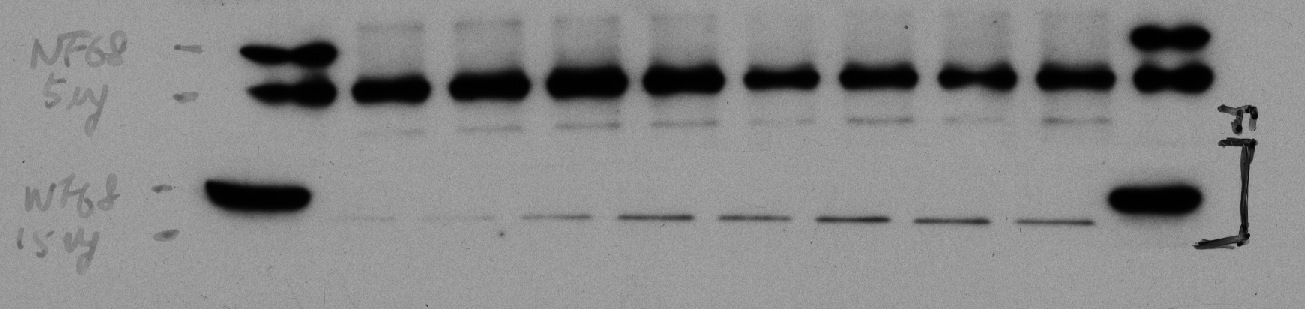


ACTIN ACTIN (Overexposed)


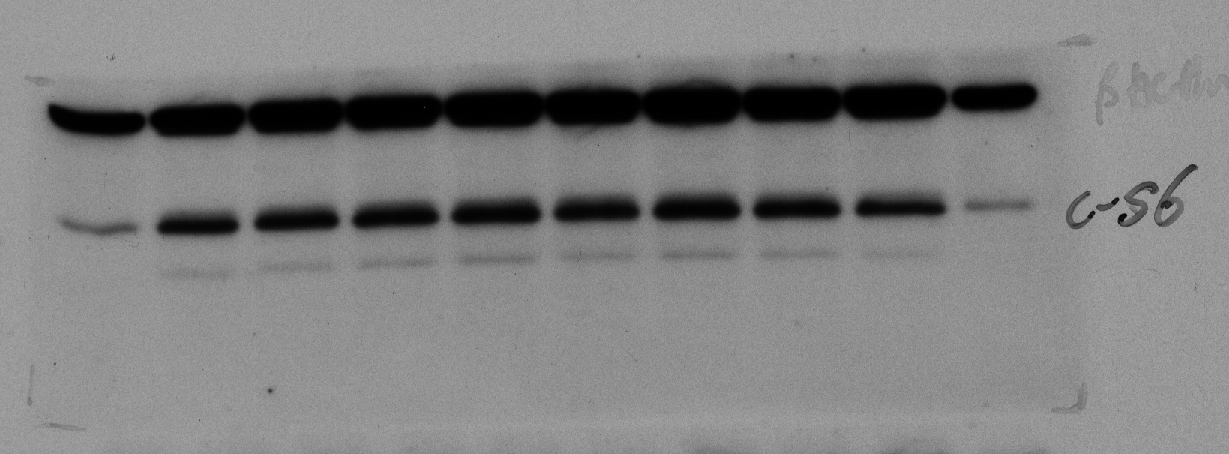


**Fig. S3C**

PTEN (Overexposed)

p-AKT p-AKT (Overexposed)


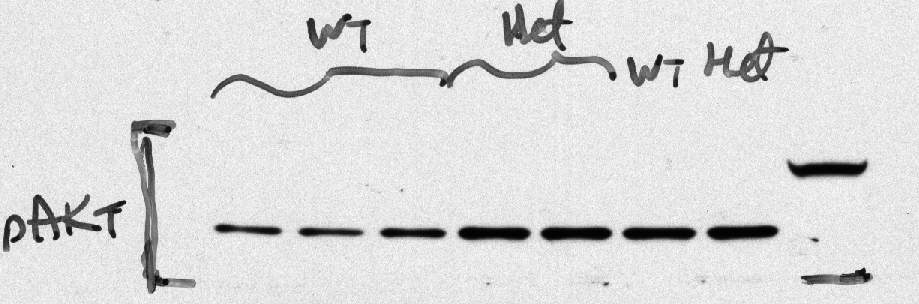


c-AKT c-AKT (Overexposed)


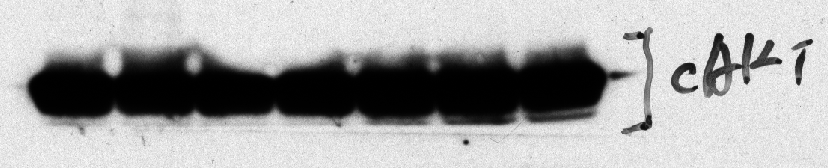


p-S6/β-actin (Overexposed)

p-S6/β-actin (Overexposed)


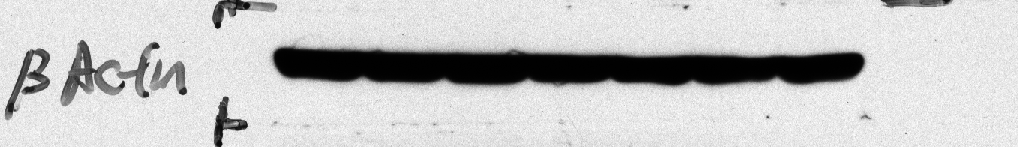

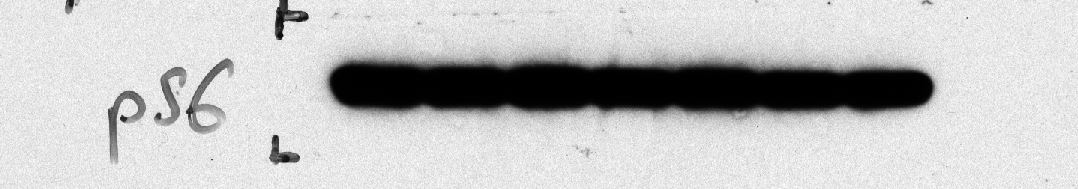


c-S6 c-S6 (Overexposed)


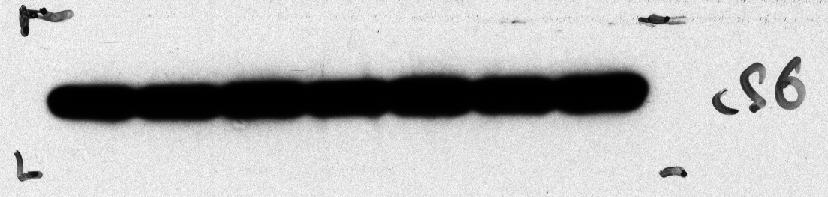


**Fig. S3E**

PTEN PTEN (Overexposed)


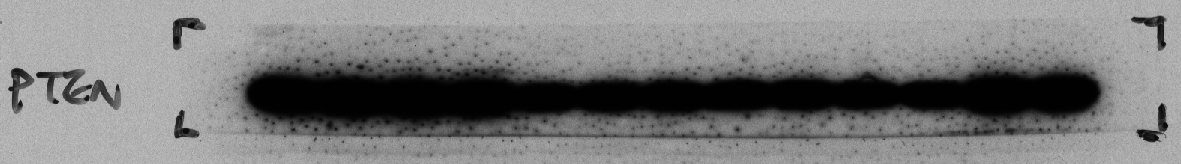


p-AKT p-AKT (Overexposed)

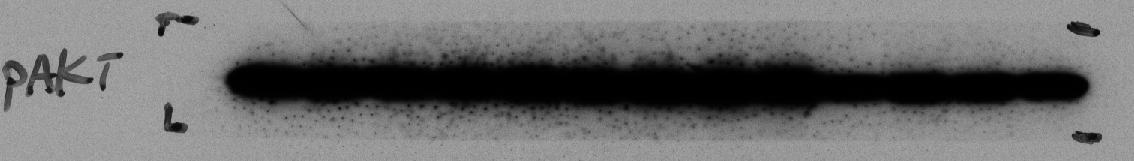

c-AKT c-AKT (Overexposed)


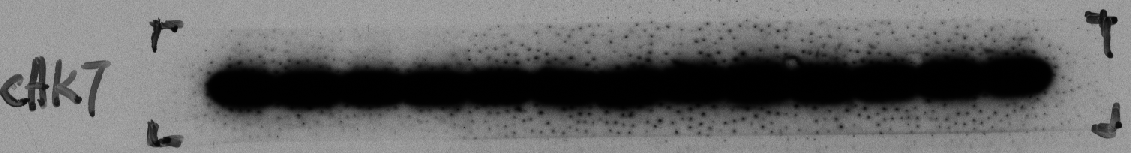


p-S6 (Overexposed)

c-S6 c-S6 (Overexposed)


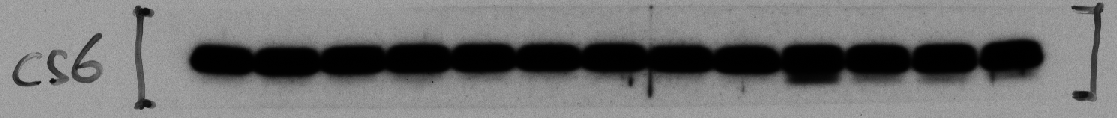


p-ERK p-ERK (Overexposed)


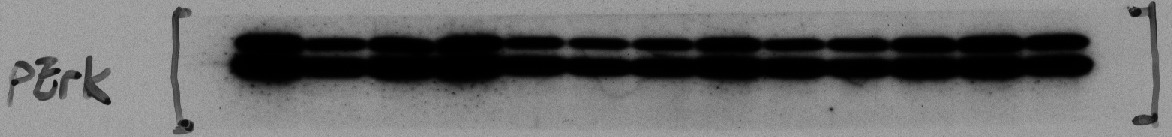


c-ERK c-ERK (Overexposed)


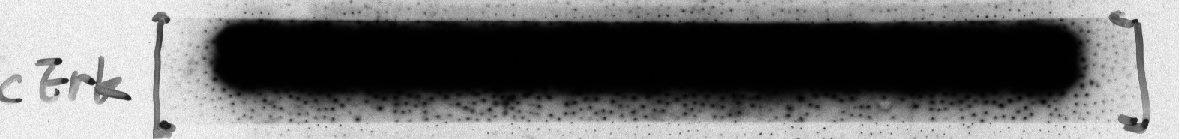


NF-68 NF68 (Long exposure)


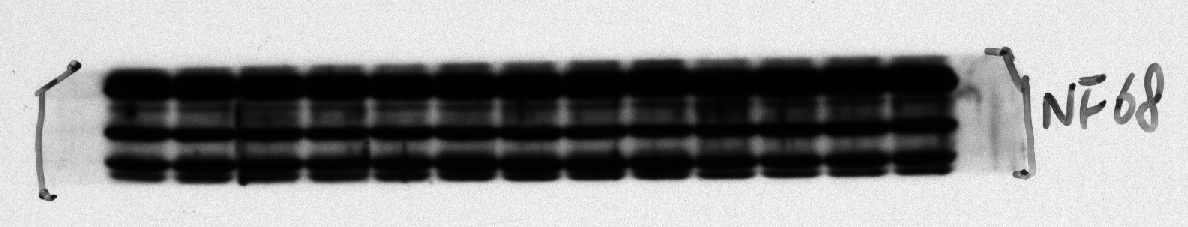


ACTIN ACTIN (Overexposed)


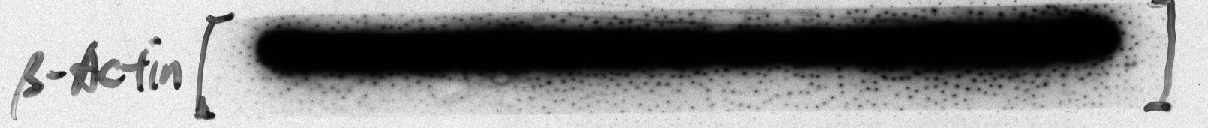


**Supplementary Fig. 6. Immunblotting of inhibitory neurons related protein in *Pten* knockout primary neuron (PCN).**

**Fig. S6**

PTEN (DIV5)/(DIV14) PTEN (DIV5)/(DIV14) (Overexposed)


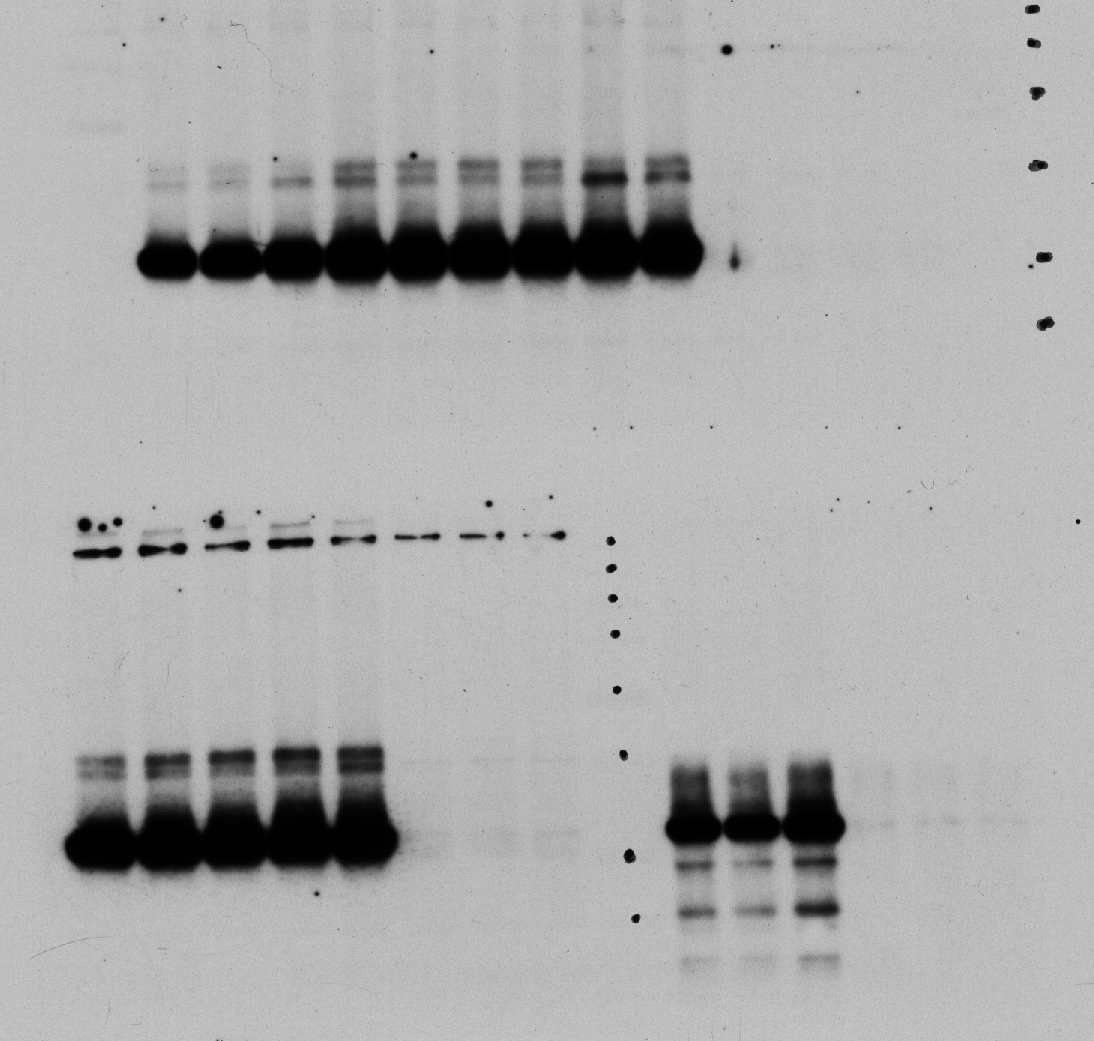


RELN (DIV5) RELN (DIV5) (Overexposed)


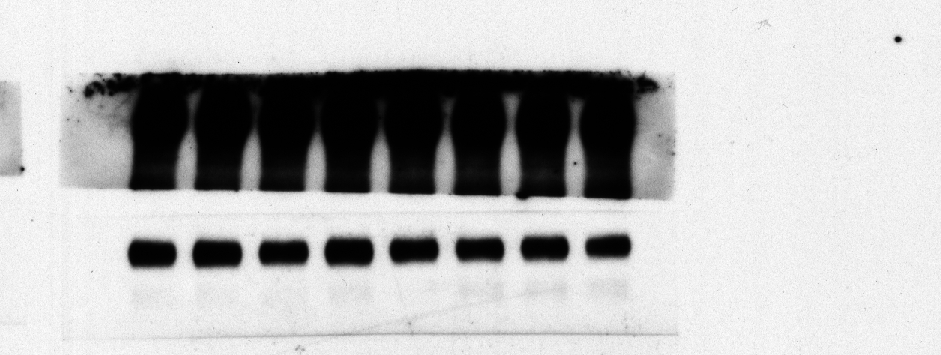


RELN (DIV14) RELN (DIV14) (Overexposed)


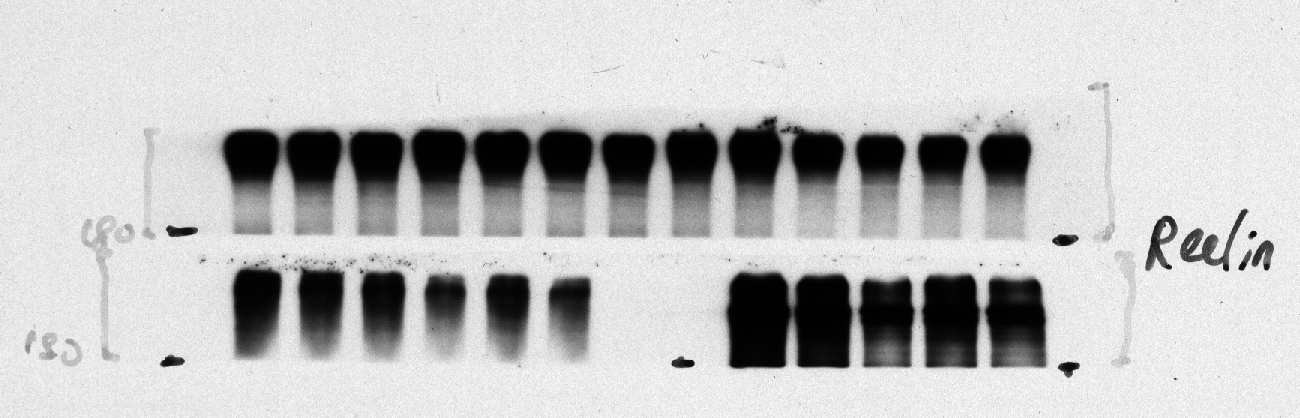


ERBB4 (DIV5) (Margin marked)

ERBB4 (DIV14) (Overexposed)

SST (DIV5) (Overexposed)

SST (DIV14) (Overexposed)

NPY (DIV5 and DIV14) (Overexposed)

ACTIN (DIV5) ACTIN (DIV5) (Overexposed)


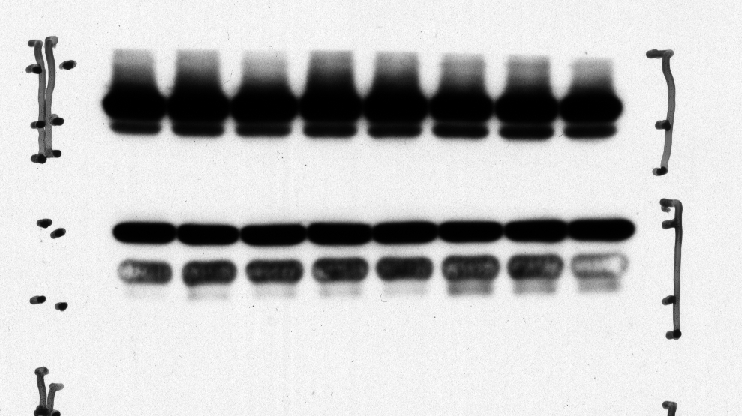


Actin (DIV14) Actin (DIV14) (Overexposed)


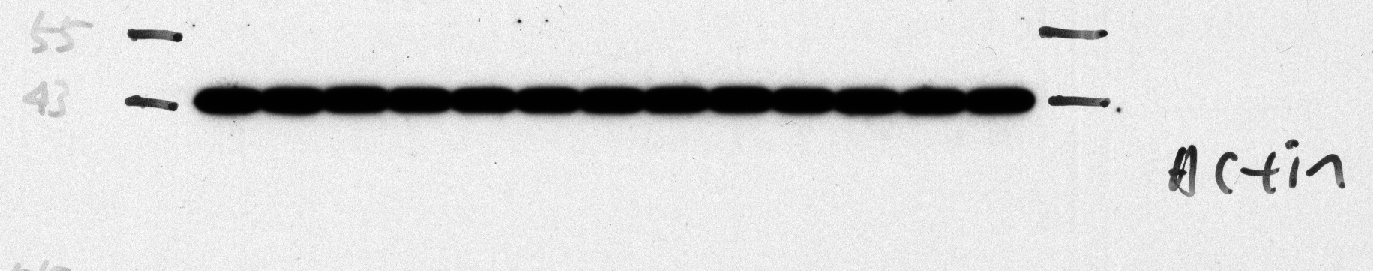

Supplement: Supplementary file 1 — Supplementary Information 1. [file 41598_2023_33869_MOESM1_ESM.docx]
